# Pre‐treatment systemic immune‐inflammation index is a useful prognostic indicator in patients with breast cancer undergoing neoadjuvant chemotherapy

**DOI:** 10.1111/jcmm.14934

**Published:** 2020-01-27

**Authors:** Li Chen, Xiangyi Kong, Zhongzhao Wang, Xiangyu Wang, Yi Fang, Jing Wang

**Affiliations:** ^1^ Department of Breast Surgical Oncology National Cancer Center/National Clinical Research Center for Cancer/Cancer Hospital Chinese Academy of Medical Sciences and Peking Union Medical College Beijing China

**Keywords:** breast cancer, neoadjuvant chemotherapy, prognosis, survival, systemic immune‐inflammation index (SII)

## Abstract

The systemic immune‐inflammation index (SII = N × P/L) based on neutrophil (N), platelet (P) and lymphocyte (L) counts is used to predict the survival of patients with malignant tumours and can fully reflect the balance between host inflammatory and immune status. This study is conducted to explore the potential prognostic significance of SII in patients with breast cancer undergoing neoadjuvant chemotherapy (NACT). A total of 262 patients with breast cancer received NACT were enrolled in this study. According to the receiver operating characteristic curve, the optimal cut‐off value of SII was divided into two groups: low SII group (<602 × 10^9^/L) and high SII group (≥602 × 10^9^/L). The associations between breast cancer and clinicopathological variables by SII were determined by chi‐squared test or Fisher's exact test. The Kaplan‐Meier plots and log‐rank test were used to determine clinical outcomes of disease‐free survival (DFS) and overall survival (OS). The prognostic value of SII was analysed by univariate and multivariate Cox proportional hazards regression models. The toxicity of NACT was accessed by National Cancer Institute Common Toxicity Criteria (NCICTC). According to univariate and multivariate Cox regression survival analyses, the results showed that the value of SII had prognostic significance for DFS and OS. The patients with low SII value had longer DFS and OS than those with high SII value (31.11 vs 40.76 months, HR: 1.075, 95% CI: 0.718‐1.610, *P* = .006; 44.47 vs 53.68 months, HR: 1.051, 95% CI: 0.707‐1.564, *P* = .005, respectively). The incidence of DFS and OS in breast cancer patients with low SII value was higher than that in those patients with high SII value in 3‐, 5‐ and 10‐year rates. The common toxicities after NACT were haematological and gastrointestinal reaction, and there were no differences by SII for the assessment of side effects of neoadjuvant chemotherapy. Meanwhile, the results also proved that breast cancer patients with low SII value and high Miller and Payne grade (MPG) survived longer than those breast cancer with high SII value and low MPG grade. In patients without lymph vessel invasion, these breast cancer patients with low SII value had better prognosis and lower recurrence rates than those with high SII value. Pre‐treatment SII with the advantage of reproducible, convenient and non‐invasive was a useful prognostic indicator for breast cancer patients undergoing neoadjuvant chemotherapy and is a promising biomarker for breast cancer on treatment strategy decisions.

## 1 INTRODUCTION

Breast cancer is one of the most frequent female malignancies and is the second cause of morbidity and mortality in women all over the world.^1^ In recent years, the incidence of breast cancer is increasing year after year, and the number of breast cancer survivors sustains growth.^2^ Moreover, 1.67 million new breast cancer cases and 522 000 deaths of breast cancer are reported worldwide per year, and the age of onset tends to be younger.^3^ In China, breast carcinoma is the leading major cause of cancer‐related mortality for females and has the highest incidence rate.^4^ The incidence of breast carcinoma is increasing rapidly in coastal developed cities, and the post‐menopausal breast cancer patients in China will reach 100/100 000 in the future.^5^ Because of the progress of early diagnosis and the improvement of treatment strategy, lots of patients have been successfully treated, and the average 5‐year survival rate is around 90%.^6^ However, approximately 20%‐25% patients are diagnosed with locally advanced breast cancer, and these patients account for early relapse and deaths.^7^, ^8^ A large number of studies have proved that surgery combined with adjuvant chemotherapy and radiotherapy can effectively improve the survival rate of patients according to the progress of early detection and treatment.^9^, ^10^, ^11^

Neoadjuvant chemotherapy (NACT) is widely used to the treatment for breast cancer and has been testified to be beneficial for breast cancer patients. The application of neoadjuvant chemotherapy in breast cancer has attracted more and more attention as a result of increasing breast‐conserving surgery rate in patients who might have required a mastectomy at initial diagnosis and decreasing tumour stage to achieve surgical operation chance and not increasing post‐operative recurrence risk.^12^, ^13^ The main purpose of NACT is to establish a therapeutic strategy based on proven therapeutic efficacy and reduce the size of tumours and improve breast‐conserving rates.^14^, ^15^ Although a number of NACT regimens have been used to treat the breast cancer, there is no internationally generally accepted NACT regimen for advanced breast carcinoma.^16^, ^17^ Therefore, it is necessary to seek some sensitive and effective indicators of breast carcinoma in order to improve the long‐term survival and provide better treatment measures.

There are some factors influencing the prognosis of the breast carcinoma, for instance, pathological stage, histologic type, molecular subtypes, tumour size, lymph vessel invasion, menopause age and so forth.^18^ Some histologic and immunologic indicators are used for assessing the prognosis of breast carcinoma. These indicators depend heavily on primary tumour samples, are often expensive, strenuous and taking a lot of time and are usually limited their use in clinical practice.^19^ To a greater extent, it is complicated when some variable factors are taken into consideration such as ER status, PR status, Ki‐67 status, HER2 status of breast carcinoma or post‐operative adjuvant treatment. Consequently, it is important to develop clinically easily reliable and accessible biomarkers to stratify the prognosis of patients with breast cancer.

In recent years, inflammation has been suggested as a critical and potentially intervenable mechanism in the pathogenesis of patients with carcinoma. Tumour associated inflammation is a fundamental part of the tumour microenvironment and could be responsible for treatment response. The changes in inflammatory cells may affect tumour carcinogenesis, development and metastasis, for example neoplastic cell proliferation, invasion migration and metastasis.^20^ The detection of peripheral blood can reflect the inflammatory state of tumours for the diagnosis and treatment of tumours. A large number of studies have clarified that the elevated inflammatory biomarkers, for instance, white blood cell (W), neutrophil (N), lymphocyte (L), monocyte (M), platelet counts (P), C‐reactive protein (CRP), as well as neutrophil‐to‐lymphocyte ratio (NLR), monocyte‐to‐lymphocyte ratio (MLR), lymphocyte‐to‐monocyte ratio (LMR) and systemic inflammation response index (SIRI), are detected in the systemic circulation and widely considered as prognostic factors for many malignant tumours.^21^, ^22^, ^23^, ^24^

An integrated and novel indicator that named systemic immune‐inflammation index (SII = N × P/L), which is based on neutrophil (N), platelet (P) and lymphocyte (L) counts, is demonstrated to be related to clinical outcomes and predict the survival time of patients with cancer.^25^, ^26^ This integrated indicator may fully reflect the balance between host inflammatory and immune status compared with NLR, LMR/MLR and PLR and other conventional haematological parameters. However, the SII has been studied rarely in breast cancer patients received NACT. As far as we are concerned, the relationship between SII and breast cancer has not been building‐up. Hence, the purpose of this study is to investigate the prognostic significance of SII in patients with breast cancer undergoing NACT.

## 2 MATERIALS AND METHODS

### 2.1 Study population

The retrospective analysis was enrolled 262 patients with advanced breast carcinoma undergoing NACT at Cancer Hospital Chinese Academy of Medical Sciences from January 1999 to December 2014. All patients were confirmed in accordance with the histology by core needle biopsy, and all cases were received NACT treatment. The details of treatment for all patients were extracted from the patients’ medical record. This study was approved by the ethics committee of Cancer Hospital Chinese Academy of Medical Sciences. It confirmed to the standards of the Declaration of Helsinki and its later amendments. All enrolled patients received the written informed consent before the study. The medical records were used to collect the clinical and demographic data of patients, and the 8th edition of the American Joint Committee Cancer Staging Manual was used to define the clinical and pathological stage.

The eligibility criteria for the patients were as follows: (a) all cases with breast carcinoma were confirmed by core needle biopsy and pathology after operation; (b) all enrolled patients were accepted surgery; (c) Karnofsky Performance Scores (KPS) ≥80 and Performance Status (Zubrod‐ECOG‐WHO, ZPS) ranged from 0 to 2 scores; (d) all enrolled patients had complete clinical recorded and follow‐up data; 4) the expected survival time over 3 months; (e) all the peripheral blood samples were obtained within one week before surgery; (f) adequate haematological, liver and renal function.

The exclusion criteria for the patients were as follows: (a) patients had accepted chemotherapy, radiotherapy, endocrine therapy and targeted therapy before beginning therapy; (b) patients with serious complications, such as infection, pneumonia; (c) patients with chronic inflammatory diseases or autoimmune disease, such as liver cirrhosis, systemic lupus erythematosus (SLE); (d) patients with distant metastasis; (e) patients were treated with blood product transfusion within one month before treatment.

### 2.2 Chemotherapy regimens

We used anthracyclines‐based and/ or taxanes‐based neoadjuvant chemotherapy regimens, and one cycle of these regimens was repeated every 3 weeks.

AC regimen: (Anthracyclines, A): 90 mg/m^2^, (Cyclophosphamide, C): 600 mg/m^2^.

On the first day, Anthracyclines (Zhejiang Hisun Pharmaceutical Co., LTD) at 90 mg/m^2^ was given by intravenous injection in 100 mL of 0.9% Normalsaline, and Cyclophosphamide (Baxter Oncology GmbH, Halle, Germany) at 600 mg/m^2^ was given by intravenous injection in 100 mL of 0.9% Normalsaline. One cycle of this regimen was repeated every 3 weeks.

ACF regimen: A: 90 mg/m^2^, C: 600 mg/m^2^, (5‐Fluorouracil, F): 500 mg/m^2^.

On the first day, Anthracyclines at 90 mg/m^2^ was given by intravenous injection in 100 mL of 0.9% Normalsaline, and Cyclophosphamide at 600 mg/m^2^ was given by intravenous injection in 100 mL of 0.9% Normalsaline. From the first day to the second day, 5‐Fluorouracil (Tianjin Jinyao Pharmaceutical Co., LTD) 500 mg/m^2^ was given by intravenous injection in 500 mL of 5% glucose above 46 h. One cycle of this regimen was repeated every 3 weeks.

CT regimen: C: 600 mg/m^2^, (Taxol, T): 175 mg/m^2^.

On the first day, Cyclophosphamide at 600 mg/m^2^ was given by intravenous injection in 100 mL of 0.9% Normalsaline, and Taxol (Jiangsu Hengrui Medicine Co., Ltd) at 175 mg/m^2^ was given by intravenous injection in 500 mL of 0.9% Normalsaline above 3 hours. One cycle of this regimen was repeated every 3 weeks.

ACT regimen: A: 90 mg/m^2^, C: 600 mg/m^2^, T: 175 mg/m^2^.

On the first day, Anthracyclines at 90 mg/m^2^ was given by intravenous injection in 100 mL of 0.9% Normalsaline, and Cyclophosphamide at 600 mg/m^2^ was given by intravenous injection in 100 mL of 0.9% Normalsaline, Taxol at 175 mg/m^2^ was given by intravenous injection in 500 mL of 0.9% Normalsaline above 3 hours. One cycle of this regimen was repeated every 3 weeks.

AT regimen: A: 90 mg/m^2^, T: 175 mg/m^2^.

On the first day, Anthracyclines at 90 mg/m^2^ was given by intravenous injection in 100 mL of 0.9% Normalsaline, and Taxol at 175 mg/m^2^ was given by intravenous injection in 500 mL of 0.9% Normalsaline above 3 hours. One cycle of this regimen was repeated every 3 weeks.

TP regimen: T: 175 mg/m^2^, (Platinum compounds, P): AUC 4‐6.

On the first day, Taxol at 175 mg/m^2^ was given by intravenous injection in 500 mL of 0.9% Normalsaline above 3 hours, and Platinum compounds (Bristol‐Myers Squibb biopharmaceutical company, Srl.) at AUC 4‐6 was given by intravenous injection in 500 mL of 0.9% Normalsaline or 500 mL of 5% glucose. One cycle of this regimen was repeated every 3 weeks.

### 2.3 Classification criteria and response evaluation

The tumour size was determined by the maximum diameter of the sample. TNM stage system was performed by the eighth edition of the American Joint Committee on Cancer (AJCC) and the Union for International Cancer Control (UICC) TNM stage classification.^27^, ^28^ Pathology type was classified as ductal carcinoma, lobular carcinoma and others. Molecular subtypes of breast cancer were divided into Luminal A, Luminal B HER2‐positive, Luminal B HER2‐negative, HER2‐enriched and Triple negative.^29^ The histologic response was estimated on the basis of Miller and Payne grade (MPG), and the MPG was categorized into five grades in line with the number of tumour cells in excision/ mastectomy specimens compared with the pre‐treatment core biopsy as follows 30:(a) grade 1, no reduction in total number of cancer cells; (b) grade 2, minor (<30%) loss of total number of cancer cells; (c) grade 3, estimated 30%‐90% reduction in total number of cancer cells; (d) grade 4, over (>90%) loss of total number of cancer cells; 5) grade 5, no infiltrating cancer (IC), or ductal carcinoma in situ. Histologic tumour grade was accessed by the Elston‐Ellis modification of Scarff‐Bloom‐Richardson grading system and based on three factors,^31^ such as: (a) the amount of gland formation; (b) the nuclear features; and (c) the mitotic activity. Each feature score was 1‐3 points and then added the score to get the final total score of 3‐9 points and was categorized into three grades by the following way 32: (a) grade I, the total score of tumours was 3‐5 points; (b) grade II, the total score of tumours was 6‐7 points; (c) grade III, the total score of tumours was 8‐9 points. The response rate was defined in the light of the Response Evaluation Criteria In Solid Tumours (RECIST) guidelines and was stratified into four groups: complete response (CR), partial response (PR), stable disease (SD) and progression of disease (PD).^33^ The sum of CR and PR were the clinical objective response rate, the sum of SD and PD were the non‐clinical response rate, and the sum of CR, PR and SD were the clinical benefit rate. Lymph vessel invasion and neural invasion were diagnosed by haematoxylin and eosin (HE) stain. The toxicity of NACT was accessed on the basis of the National Cancer Institute Common Toxicity Criteria (NCICTC).^34^

### 2.4 Peripheral venous blood parameters

Peripheral venous blood samples were taken within 1 week after breast cancer diagnosis and before neoadjuvant chemotherapy. The sterile EDTA tube was used to collect the blood samples. The XE‐2100 haematology analyser (Sysmex, Kobe, Japan) was used to analyse the haematological parameters.

### 2.5 Follow‐up

All enrolled patients had post‐operative follow‐up in inpatients or outpatients every 3 months for the 1st to 2nd year, every 6 months for the 3rd to 5th year after surgery, then annually every year and until death.^35^ Follow‐up assessments included laboratory tests (routine blood test, blood biochemical), physical examination (breast and lymph node palpation), breast ultrasonography, liver ultrasound, mammography and other suitable examinations. Disease‐free survival (DFS) referred to the time from the date of operation to the date of local recurrence or distant metastases, death from any reason or final follow‐up. Overall survival (OS) referred to the time from the date of operation to the date of death from any reason or final follow‐up.

### 2.6 Statistical analysis

Statistical analyses were conducted by SPSS software (version 17.0) and GraphPad prism software (version 5.0). The optimal cut‐off value was accessed by the receiver operating characteristic curve (ROC) analyses. The clinicopathologic categorical variables were performed as frequencies and percentages (%), and the associations between breast carcinoma and clinicopathological variables were evaluated by chi‐squared test or Fisher's exact test. The Kaplan‐Meier product limit estimator method was to determine the survival time of DFS and OS, and the association between breast carcinoma and survival was analysed by the Kaplan‐Meier plots and log‐rank test. The Cox proportional hazards regression model was used to examine the independent prognostic factors. *P* < .05 was considered to be statistical significance.

## 3 RESULTS

### 3.1 Demographic and clinicopathologic characteristics of all breast cancer patients

Two hundred and sixty two patients with breast carcinoma were included in this study. The clinicopathological characteristics of patients with breast cancer were shown in Table 1. The ROC analysis was intended for evaluating the optimal cut‐off value of the SII (602 × 10^9^/L). Hence, the patients were categorized into two groups: low SII group (<602 × 10^9^/L) and high SII group (≥602 × 10^9^/L). All cases were females, and the median age was 48 years, with the range from 27 to 73 years. There were 156 patients (59.5%) in low SII group and 106 patients (40.5%) in high SII group. The median body mass index (BMI) was 24.50, and the range was 18.03‐39.06. All patients with breast cancer received anthracyclines‐based and taxanes‐based neoadjuvant chemotherapy regimens (27 patients received the AC/ACF regimen, 29 patients received the CT/ACT regimen, 121 patients received the AT regimen, 75 patients received TP regimen, and 10 patients received other regimens, such as FP, T, F, TF, EV regimen. All 262 patients underwent surgery, and 221 cases underwent mastectomy, 41 cases underwent breast‐conserving surgery. In terms of histologic pathology, 251 patients had ductal carcinoma, 4 patients had lobular carcinoma, and 7 patients had other pathology (metaplastic carcinoma, mucinous carcinoma of breast). The clinicopathological characteristics were similar between the two groups (Table 1). In our study, patients with low SII value were significantly associated with menopause (*χ* ^2^ = 5.723, *P* = .016), US‐LNM (*χ* ^2^ = 4.331, *P* = .037).

**Table 1:** Demographic and clinicopathologic characteristics of all enrolled breast cancer patients

Parameters Cases (n)	262	Low SII < 602	High SII ≥ 602	*χ* ^2^	*P* value
		156 (59.5%)	106 (40.5%)		
Age (years)					
<48	138 (52.7%)	77 (49.4%)	61 (57.6%)	1.697	.193
≥48	124 (47.3%)	79 (50.6%)	45 (42.4%)		
Marital status					
Married	243 (92.7%)	146 (93.6%)	97 (91.5%)	1.883	.757
Unmarried	9 (3.4%)	6 (3.9%)	3 (2.8%)		
Divorce	7 (2.7%)	3 (1.9%)	4 (3.8%)		
Widowhood	3 (1.2%)	1 (0.6%)	2 (1.9%)		
Occupation					
Mental worker	165 (63.0%)	100 (64.1%)	65 (61.3%)	0.807	.668
Manual worker	35 (13.4%)	22 (14.1%)	13 (12.3%)		
Others	62 (23.6%)	34 (21.8%)	28 (26.4%)		
BMI					
<24.50	148 (56.5%)	84 (53.9%)	64 (60.4%)	1.095	.295
≥24.50	114 (43.5%)	72 (46.1%)	42 (39.6%)		
Menopause					
No	160 (61.1%)	86 (55.1%)	74 (69.8%)	5.723	.016
Yes	102 (38.9%)	70 (44.9%)	32 (30.2%)		
ABO blood type					
A	84 (32.1%)	53 (34.0%)	31 (29.3%)	3.463	.483
B	78 (29.7%)	46 (29.5%)	32 (30.2%)		
O	73 (27.9%)	38 (24.4%)	35 (33.0%)		
AB	27 (20.3%)	19 (12.1%)	8 (7.5%)		
Tumour site					
Right	126 (48.1%)	77 (49.4%)	49 (46.2%)	0.248	.618
Left	136 (51.9%)	79 (50.6%)	57 (53.8%)		
Primary tumour site					
Upper outer quadrant	166 (63.4%)	95 (60.9%)	71 (67.0%)	3.351	.501
Lower outer quadrant	21 (8.0%)	13 (8.3%)	8 (7.6%)		
Lower inner quadrant	12 (4.6%)	8 (5.1%)	4 (3.7%)		
Upper inner quadrant	38 (14.5%)	27 (17.3%)	11 (10.4%)		
Central	25 (9.5%)	13 (8.4%)	12 (11.3%)		
US‐Tumour size					
≤2 cm	95 (36.3%)	62 (39.7%)	33 (31.1%)	2.043	.360
>2 and < 5cm	138 (52.6%)	78 (50.0%)	60 (56.6%)		
≥5cm	29 (11.1%)	16 (10.3%)	13 (12.3%)		
US‐LNM					
No	156 (59.5%)	101 (64.7%)	55 (51.9%)	4.331	.037
Yes	106 (40.5%)	55 (35.3%)	51 (48.1%)		
US‐BIRADS classification					
4	36 (13.8%)	20 (12.8%)	16 (15.0%)	1.450	.484
5	103 (39.3%)	58 (37.2%)	45 (42.5%)		
6	123 (46.9%)	78 (50.0%)	45 (42.5%)		
Clinical stage					
Clinical T stage					
T1	47 (17.9%)	31 (19.9%)	16 (15.1%)	4.788	.310
T2	117 (44.7%)	75 (48.1%)	42 (39.6%)		
T3	66 (25.2%)	34 (21.8%)	32 (30.2%)		
T4	32 (12.2%)	16 (10.2%)	16 (15.1%)		
Clinical N stage					
N0	48 (18.3%)	29 (18.6%)	19 (17.9%)	3.038	.552
N1	89 (34.0%)	58 (37.2%)	31 (29.3%)		
N2	77 (29.4%)	45 (28.9%)	32 (30.2%)		
N3	48 (18.3%)	24 (15.3%)	24 (22.6%)		
Clinical TNM stage					
II	107 (40.8%)	69 (44.2%)	38 (35.9%)	1.835	.176
III	155 (59.2%)	87 (55.8%)	68 (64.1%)		
Type of surgery					
Mastectomy	221 (84.4%)	130 (83.3%)	91 (85.9%)	0.302	.582
Breast‐conserving surgery	41 (15.6%)	26 (16.7%)	15 (14.1%)		
Tumour size					
≤2 cm	117 (44.7%)	68 (43.6%)	49 (46.2%)	1.124	.570
>2 and < 5cm	120 (45.8%)	75 (48.1%)	45 (42.5%)		
≥5cm	25 (9.5%)	13 (8.3%)	12 (11.3%)		
Histologic type					
Ductal	251 (95.8%)	148 (94.9%)	103 (97.2%)	2.176	.337
Lobular	4 (1.5%)	2 (1.3%)	2 (1.9%)		
Others	7 (2.7%)	6 (3.8%)	1 (0.9%)		
Histologic grade					
I	74 (28.2%)	41 (26.3%)	33 (31.1%)	1.263	.532
II	136 (51.9%)	81 (51.9%)	55 (51.9%)		
III	52 (19.9%)	34 (21.8%)	18 (17.0%)		
Pathological TNM classification					
Pathological T stage					
Tis/T0	42 (16.0%)	25 (16.0%)	17 (16.0%)	4.331	.363
T1	101 (38.6%)	60 (38.5%)	41 (38.7%)		
T2	82 (31.3%)	54 (34.6%)	28 (26.4%)		
T3	25 (9.5%)	12 (7.7%)	13 (12.3%)		
T4	12 (4.6%)	5 (3.2%)	7 (6.6%)		
Pathological N stage					
N0	98 (37.4%)	58 (37.2%)	40 (37.7%)	3.320	.506
N1	51 (19.5%)	33 (21.2%)	18 (17.0%)		
N2	40 (15.3%)	27 (17.3%)	13 (12.3%)		
N3	73 (27.8%)	38 (24.3%)	35 (33.0%)		
Pathological TNM stage					
Tis/T0	34 (13.0%)	18 (11.5%)	16 (15.1%)	2.449	.654
I	47 (17.9%)	31 (19.9%)	16 (15.1%)		
II	59 (22.5%)	38 (24.4%)	21 (19.8%)		
III	122 (46.6%)	69 (44.2%)	53 (50.0%)		
Total lymph nodes					
<21	120 (45.8%)	72 (46.2%)	48 (45.3%)	0.019	.890
≥21	142 (54.2%)	84 (53.8%)	58 (54.7%)		
Positive lymph nodes					
0	97 (37.0%)	57 (36.5%)	40 (37.7%)	0.630	.730
<6	71 (27.1%)	45 (28.9%)	26 (24.5%)		
≥6	94 (35.9%)	54 (34.6%)	40 (37.8%)		
Total axillary lymph nodes					
<20	118 (45.0%)	74 (47.4%)	44 (41.5%)	0.896	.344
≥20	144 (55.0%)	82 (52.6%)	62 (58.5%)		
Positive axillary lymph nodes					
0	99 (37.8%)	59 (37.8%)	40 (37.7%)	0.676	.713
<5	63 (24.1%)	40 (25.6%)	23 (21.7%)		
≥5	100 (38.1%)	57 (36.6%)	43 (40.6%)		
Post‐operative complications					
No	253 (96.6%)	151 (96.8%)	102 (96.2%)	0.061	.804
Yes	9 (3.4%)	5 (3.2%)	4 (3.8%)		
Post‐operative radiotherapy					
No	61 (23.3%)	38 (24.4%)	23 (21.7%)	0.250	.617
Yes	201 (76.7%)	118 (75.6%)	83 (78.3%)		
Post‐operative endocrine therapy					
No	130 (49.6%)	75 (48.1%)	55 (51.9%)	0.366	.545
Yes	132 (50.4%)	81 (51.9%)	51 (48.1%)		
Post‐operative targeted therapy					
No	189 (72.1%)	119 (76.3%)	70 (66.0%)	3.295	.069
Yes	73 (27.9%)	37 (23.7%)	36 (34.0%)		

### 3.2 Relationships between SII and haematological parameters

In our study, the optimal cut‐off value of SII was decided as 602 × 10^9^/L, which was calculated considering the maximum (sensitivity + specificity) point of the ROC curve. ROC curve was analysed with OS data of all enrolled patients. The cut‐off value of ALT, AST, LDH, IgA, IgG, IgM, ALB, CRP, CA125, CA153, CEA, D‐D, FDP, W, red blood cell (R), haemoglobin (Hb), N, M, P and L, NLR, MLR, PLR were determined by its median value, and the cut‐off values were 23, 23, 190 U/L, 2.30, 11.67, 1.27, 44.00 g/L, 1.10 mg/dL, 27.73 U/mL, 21.86 U/mL, 3.52 ng/ml, 0.83 mg/L FEU, 2.01 µg/mL, and 6.00 × 10^9^/L, 4.34 × 10^9^/L, 128.00 × 10^9^/L, 3.83 × 10^9^/L, 1.67 × 10^9^/L, 0.34 × 10^9^/L, 244.00 × 10^9^/L, 2.50, 0.22, 160.00, respectively. A low SII was significantly related to ALB (*P* = .001), CRP (*P* = .042), CA125 (*P* = .044), W (*P* < .001), R (*P* = .024), Hb (*P* = .026), N (*P* < .001), P (*P* = .001), NLR (*P* < .001), MLR (*P* < .001), PLR (*P* < .001). (Table 2).

**Table 2:** Relationships between SII and haematological parameters

Parameters Cases (n)	262	Low SII < 602	High SII ≥ 602	*χ* ^2^	*P* value
		156 (59.5%)	106 (40.5%)		
ALT					
<23	183 (69.9%)	114 (73.1%)	69 (65.1%)	1.910	.167
≥23	79 (30.1%)	42 (26.9%)	37 (34.9%)		
AST					
<23	182 (69.5%)	108 (69.2%)	74 (69.8%)	0.010	.920
≥23	80 (30.5%)	48 (30.8%)	32 (30.2%)		
LDH					
<190	174 (66.4%)	109 (69.9%)	65 (61.3%)	2.069	.150
≥190	88 (33.6%)	47 (30.1%)	41 (38.7%)		
IgA					
<2.30	136 (51.9%)	85 (54.5%)	51 (48.1%)	1.027	.311
≥2.30	126 (48.1%)	71 (45.5%)	55 (51.9%)		
IgG					
<11.67	141 (53.8%)	88 (56.4%)	53 (50.0%)	1.043	.307
≥11.67	121 (46.2%)	68 (43.6%)	53 (50.0%)		
IgM					
<1.27	160 (61.1%)	99 (63.5%)	61 (57.6%)	0.927	.335
≥1.27	102 (38.9%)	57 (36.5%)	45 (42.4%)		
ALB					
<44.00	98 (37.4%)	71 (45.5%)	27 (25.5%)	10.827	.001
≥44.00	164 (62.6%)	85 (54.5%)	79 (74.5%)		
CRP					
<1.10	226 (86.3%)	129 (82.7%)	97 (91.5%)	4.140	.042
≥1.10	36 (13.7%)	27 (17.3%)	9 (8.5%)		
CA125					
<27.73	224 (85.5%)	139 (89.1%)	85 (80.2%)	4.044	.044
≥27.73	38 (14.5%)	17 (10.9%)	21 (19.8%)		
CA153					
<21.86	209 (79.8%)	130 (83.3%)	79 (74.5%)	3.032	.081
≥21.86	53 (20.2%)	26 (16.7%)	27 (25.5%)		
CEA					
<3.52	211 (80.5%)	130 (83.3%)	81 (76.4%)	1.927	.165
≥3.52	51 (19.5%)	26 (16.7%)	25 (23.6%)		
D‐D					
<0.83	218 (83.2%)	130 (83.3%)	88 (83.0%)	0.005	.947
≥0.83	44 (16.8%)	26 (16.7%)	18 (17.0%)		
FDP					
<2.01	156 (59.5%)	92 (59.0%)	64 (60.4%)	0.052	.820
≥2.01	106 (40.5%)	64 (41.0%)	42 (39.6%)		
White blood cell (W)					
<6.00	133 (50.8%)	101 (64.7%)	32 (30.2%)	30.152	<.001
≥6.00	129 (49.2%)	55 (35.3%)	74 (69.8%)		
Red blood cell (R)					
<4.34	116 (44.3%)	78 (50.0%)	38 (35.9%)	5.122	.024
≥4.34	146 (55.7%)	78 (50.0%)	68 (64.1%)		
Haemoglobin (Hb)					
<128.00	108 (41.2%)	73 (46.8%)	35 (33.0%)	4.943	.026
≥128.00	154 (58.8%)	83 (53.2%)	71 (67.0%)		
Neutrophil (N)					
<3.83	134 (51.2%)	115 (73.7%)	19 (17.9%)	78.629	<.001
≥3.83	128 (48.8%)	41 (26.3%)	87 (82.1%)		
Lymphocyte (L)					
<1.67	143 (54.6%)	78 (50.0%)	65 (61.3%)	3.263	.071
≥1.67	119 (45.4%)	78 (50.0%)	41 (38.7%)		
Monocyte (M)					
<0.34	133 (50.8%)	86 (55.1%)	47 (44.3%)	2.939	.086
≥0.34	129 (49.2%)	70 (44.9%)	59 (55.7%)		
Platelet (P)					
<244.00	136 (51.9%)	94 (60.3%)	42 (39.6%)	10.764	.001
≥244.00	126 (48.1%)	62 (39.7%)	64 (60.4%)		
NLR					
<2.50	160 (61.1%)	137 (87.8%)	23 (21.7%)	116.067	<.001
≥2.50	102 (38.9%)	19 (12.2%)	83 (78.3%)		
MLR					
<0.22	152 (58.0%)	107 (68.6%)	45 (42.5%)	17.703	<.001
≥0.22	110 (42.0%)	49 (31.4%)	61 (57.5%)		
PLR					
<160.00	158 (60.3%)	122 (78.2%)	36 (34.0%)	51.609	<.001
≥160.00	104 (39.7%)	34 (21.8%)	70 (66.0%)		

### 3.3 Association of SII and neoadjuvant chemotherapy or post‐operative chemotherapy

All enrolled patients received NACT treatment, and the median number of pre‐operative chemotherapy was six times, and 27 patients received the AC/ACF regimen, 29 patients received the CT/ACT regimen, 121 patients received the AT regimen, 75 patients received TP regimen, and 10 patients received other regimens, such as FP, T, F, TF, EV regimen. However, only 116 patients were received post‐operative chemotherapy, and the median number of post‐operative chemotherapies was 4 times, and 146 patients were not received post‐operative chemotherapy. 18 cases were treated with AC/ACF regimen, 17 cases were treated with CT/ACT regimen, 27 cases were treated with AT regimen, 21 cases were treated with TP regimen, and 33 patients were treated with other regimens, such as C, CTF, CTP, A, AF, AMF, AP, F, FP, T, TF, V, VP regimen. The clinical objective response rate (CR + PR) was 61.4%, and the clinical benefit rate (CR + PR+SD) was 97.3%, and non‐clinical response rate (SD + PD) was 38.6%. We also used the MPG system to evaluate the pathological response, and the grade 1 rate was 5.0%, the grade 2 rate was 29.8%, the grade 3 rate was 39.3%, the grade 4 rate was 10.3%, and the grade 5 rate was 15.6%. The pathological response of pCR rate was 20.6%, and the pathological response of non‐pCR rate was 79.4%. (Table 3).

**Table 3:** Association of SII and neoadjuvant chemotherapy or post‐operative chemotherapy

Parameters Cases (n)	262	Low SII < 602	High SII ≥ 602	*χ* ^2^	*P* value
		156 (59.5%)	106 (40.5%)		
Neoadjuvant chemotherapy regimen					
AC/ACF	27 (10.3%)	18 (11.5%)	9 (8.5%)	7.654	.105
CT/ACT	29 (11.1%)	20 (12.8%)	9 (8.5%)		
AT	121 (46.2%)	74 (47.4%)	47 (44.3%)		
TP	75 (28.6%)	36 (23.2%)	39 (36.8%)		
Others	10 (3.8%)	8 (5.1%)	2 (1.9%)		
Chemotherapy times					
<6	85 (32.4%)	56 (35.9%)	29 (27.4%)	2.100	.147
≥6	177 (67.6%)	100 (64.1%)	77 (72.6%)		
Response					
CR	5 (1.9%)	5 (3.2%)	0 (0.0%)	6.675	.154
PR	156 (59.5%)	85 (54.5%)	71 (67.0%)		
SD	94 (35.9%)	62 (39.7%)	32 (30.2%)		
PD	7 (2.7%)	4 (2.6%)	3 (2.8%)		
Miller and Payne grade					
1	13 (5.0%)	5 (3.2%)	8 (7.6%)	3.902	.419
2	78 (29.8%)	51 (32.7%)	27 (25.4%)		
3	103 (39.3%)	62 (39.7%)	41 (38.7%)		
4	27 (10.3%)	15 (9.6%)	12 (11.3%)		
5	41 (15.6%)	23 (14.8%)	18 (17.0%)		
Pathological response					
pCR	54 (20.6%)	30 (19.2%)	24 (22.6%)	0.449	.503
non‐pCR	208 (79.4%)	126 (80.8%)	82 (77.4%)		
Post‐operative chemotherapy regimen					
0	146 (55.7%)	86 (55.1%)	60 (56.6%)	3.765	.584
AC/ACF	18 (6.9%)	8 (5.1%)	10 (9.4%)		
CT/ACT	17 (6.5%)	10 (6.4%)	7 (6.6%)		
AT	27 (10.3%)	15 (9.6%)	12 (11.4%)		
TP	21 (8.0%)	14 (9.0%)	7 (6.6%)		
Others	33 (12.6%)	23 (14.8%)	10 (9.4%)		
Post‐operative chemotherapy times					
0	146 (55.7%)	86 (55.1%)	60 (56.6%)	0.531	0.767
<4	41 (15.7%)	23 (14.7%)	18 (17.0%)		
≥4	75 (28.6%)	47 (30.2%)	28 (26.4%)		

### 3.4 Correlation between SII and breast cancer molecular subtypes

All breast cancer patients enrolled in this study were confirmed by core needle biopsy prior to NAC. ER receptor and PR receptor were decided as positive when at least 1% of tumour cells were stained by nuclei. Breast cancer with 0 or 1+ on immunohistochemical staining was considered negative for HER2 and with 3+ on immunohistochemical staining was regarded as HER2 overexpression. However, HER2 with 2+ on immunohistochemical staining was further evaluated by fluorescence in situ hybridization, and patient with HER2/CEP17 ≥ 2.0 was also determined to show HER2 overexpression. Ki‐67 labelling index ≤14% tumour cells with nuclear staining was considered to be negative, and Ki‐67 labelling index >14% tumour cells with nuclear staining was considered to be positive. Before neoadjuvant chemotherapy, 8 patients were Luminal A subtype, 27 patients were Luminal B HER2‐positive subtype, 98 patients were Luminal B HER2‐negative subtype, 62 patients were HER2‐enriched subtype, and 67 patients were Triple negative subtype, respectively. And 108 patients were ER negative, 154 patients were ER positive; 129 patients were PR negative, 133 patients were PR positive; 168 patients were HER2 negative, 94 patients were HER2 positive; 60 patients were Ki‐67 negative, 202 patients were Ki‐67 positive, respectively. After post‐operative immunohistochemical pathology, 9 patients were Luminal A subtype, 25 patients were Luminal B HER2‐positive subtype, 94 patients were Luminal B HER2‐negative subtype, 66 patients were HER2‐enriched subtype, and 68 patients were Triple negative subtype, respectively. And 122 patients were ER negative, 140 patients were ER positive; 131 patients were PR negative, 131 patients were PR positive; 173 patients were HER2 negative, 89 patients were HER2 positive; 92 patients were Ki‐67 negative, 170 patients were Ki‐67 positive, respectively. Moreover, some other indicators were detected, such as AR, CK5/6, E‐cad, P53, and TOP2A. And 164 cases were AR negative, 98 cases were AR positive; 228 cases were CK5/6 negative, 34 cases were CK5/6 positive; 109 patients were E‐cad negative, 153 patients were E‐cad positive; 198 cases were EGFR negative, 64 cases were EGFR positive; 132 cases were P53 negative, 130 cases were P53 positive; 107 cases were TOP2A negative, 155 cases were TOP2A positive, respectively. We also found that 100 patients with breast cancer were diagnosed with lymph vessel invasion, and 65 patients with breast cancer were diagnosed with neural invasion. A low SII was significantly related to core needle biopsy ER status (*χ* ^2^ = 5.662, *P* = .017), post‐operative pathology IHC HER2 status (*χ* ^2^ = 4.512, *P* = .034). (Table 4).

**Table 4:** Correlation between SII and breast cancer molecular subtypes

Parameters Cases (n)	262	Low SII < 602	High SII ≥ 602	*χ* ^2^	*P* value
		156 (59.5%)	106 (40.5%)		
Core needle biopsy					
Molecular subtype					
Luminal A	8 (3.1%)	6 (3.8%)	2 (1.9%)	4.306	.366
Luminal B HER2+	27 (10.3%)	15 (9.6%)	12 (11.3%)		
Luminal B HER2‐	98 (37.4%)	63 (40.4%)	35 (33.0%)		
HER2 enriched	62 (23.7%)	31 (19.9%)	31 (29.3%)		
Triple negative	67 (25.5%)	41 (26.3%)	26 (24.5%)		
ER status					
Negative	108 (41.2%)	55 (35.3%)	53 (50.0%)	5.662	.017
Positive	154 (58.8%)	101 (64.7%)	53 (50.0%)		
PR status					
Negative	129 (49.2%)	78 (50.0%)	51 (48.1%)	0.090	.764
Positive	133 (50.8%)	78 (50.0%)	55 (51.9%)		
HER2 status					
Negative (0‐‐++)	168 (64.1%)	107 (68.6%)	61 (57.6%)	3.345	.067
Positive (+++)	94 (35.9%)	49 (31.4%)	45 (42.4%)		
Ki‐67 status					
Negative (≤14%)	60 (22.9%)	40 (25.6%)	20 (18.9%)	1.640	.200
Positive (>14%)	202 (77.1%)	116 (74.4%)	86 (81.1%)		
Post‐operative pathology (IHC)					
Molecular subtype					
Luminal A	9 (3.4%)	7 (4.5%)	2 (1.9%)	8.486	.075
Luminal B HER2+	25 (9.5%)	13 (8.3%)	12 (11.3%)		
Luminal B HER2‐	94 (35.9%)	63 (40.4%)	31 (29.3%)		
HER2 enriched	66 (25.2%)	31 (19.9%)	35 (33.0%)		
Triple negative	68 (26.0%)	42 (26.9%)	26 (24.5%)		
ER status					
Negative	122 (46.6%)	65 (41.7%)	57 (53.8%)	3.718	.053
Positive	140 (53.4%)	91 (58.3%)	49 (46.2%)		
PR status					
Negative	131 (50.0%)	76 (48.7%)	55 (51.9%)	0.253	.615
Positive	131 (50.0%)	80 (51.3%)	51 (48.1%)		
HER2 status					
Negative (0−−++)	173 (66.0%)	111 (71.2%)	62 (58.5%)	4.512	.034
Positive (+++)	89 (34.0%)	45 (28.8%)	44 (41.5%)		
Ki‐67 status					
Negative (≤14%)	92 (35.1%)	56 (35.9%)	36 (34.0%)	0.104	.747
Positive (>14%)	170 (64.9%)	100 (64.1%)	70 (66.0%)		
AR status					
Negative	164 (62.6%)	103 (66.0%)	61 (57.6%)	1.938	.163
Positive	98 (37.4%)	53 (34.0%)	45 (42.4%)		
CK5/6 status					
Negative	228 (87.0%)	137 (87.8%)	91 (85.9%)	0.217	.641
Positive	34 (13.0%)	19 (12.2%)	15 (14.1%)		
E‐cad status					
Negative	109 (41.6%)	69 (44.2%)	40 (37.7%)	1.096	.295
Positive	153 (58.4%)	87 (55.8%)	66 (62.3%)		
EGFR status					
Negative	198 (75.6%)	119 (76.3%)	79 (74.5%)	0.105	.745
Positive	64 (24.4%)	37 (23.7%)	27 (25.5%)		
P53 status					
Negative	132 (50.4%)	81 (51.9%)	51 (48.1%)	0.366	.545
Positive	130 (49.6%)	75 (48.1%)	55 (51.9%)		
TOP2A status					
Negative	107 (40.8%)	62 (39.7%)	45 (42.5%)	0.192	.661
Positive	155 (59.2%)	94 (60.3%)	61 (57.5%)		
Lymph vessel invasion					
Negative	162 (61.8%)	98 (62.8%)	64 (60.4%)	0.160	.690
Positive	100 (38.2%)	58 (37.2%)	42 (39.6%)		
Neural invasion					
Negative	197 (75.2%)	121 (77.6%)	76 (71.7%)	1.164	.281
Positive	65 (24.8%)	35 (22.4%)	30 (28.3%)		

### 3.5 Correlation between SII and side effects of chemotherapy

In our study, the side effects of neoadjuvant chemotherapy for two cycles were evaluated and analysed by NCICTC. We also found that were the haematological and gastrointestinal reaction were the common toxicities after NACT. Decreased appetite, nausea, vomiting, diarrhoea, mouth ulcers, alopecia and peripheral neurotoxicity were recorded in 92.4% (242/262), 94.7% (248/262), 62.6% (164/262), 9.5% (25/262), 3.8% (10/262), 57.6% (151/262), and 8.8% (23/262), respectively. Moreover, the toxicities by the NCICTC indicated that grade 0 anaemia, leukopenia, neutropenia, thrombocytopenia, gastrointestinal reaction and myelosuppression were recorded in 37.4% (98/262), 29.4% (77/262), 27.5% (72/262), 67.9% (178/262), 3.4% (9/262) and 21.0% (55/262); grade 1/2 anaemia, leukopenia, neutropenia, thrombocytopenia, gastrointestinal reaction and myelosuppression were recorded in 61.8% (162/262), 40.5% (106/262), 39.7% (104/262), 30.9% (81/262), 95.4% (250/262) and 38.5% (101/262); grade 3/4 anaemia, leukopenia, neutropenia, thrombocytopenia, gastrointestinal reaction and myelosuppression were recorded in 0.8% (2/262), 30.1% (79/262), 32.8% (86/262), 1.2% (3/262), 1.2% (3/262) and 40.5% (106/262), respectively. There were no chemotherapy‐related deaths occurred in this study. Further studies on the side effects of NACT evaluation in SII proved that there were no differences using the SII in decreased appetite, nausea, vomiting, diarrhoea, mouth ulcers, alopecia, peripheral neurotoxicity (*χ* ^2^ = 0.983, 0.138, 1.281, 0.653, 1.806, 1.682, 0.337; *P* > .05), and anaemia, leukopenia, neutropenia, thrombocytopenia, gastrointestinal reaction and myelosuppression (*χ* ^2^ = 1.654, 0.694, 1.767, 2.605, 2.285, 0.337; *P* > .05), respectively. (Table 5).

**Table 5:** Main toxicities according to NCI‐CTC scale of the patients with breast cancer undergoing neoadjuvant chemotherapy

Parameters Cases (n)	262	Low SII < 602	High SII ≥ 602	*χ* ^2^	*P* value
		156 (59.5%)	106 (40.5%)		
Decreased appetite					
No	20 (7.6%)	14 (9.0%)	6 (5.7%)	0.983	.321
Yes	242 (92.4%)	142 (91.0%)	100 (94.3%)		
Nausea					
No	14 (5.3%)	9 (5.8%)	5 (4.7%)	0.138	0.710
Yes	248 (94.7%)	147 (94.2%)	101 (95.3%)		
Vomiting					
No	98 (37.4%)	54 (34.6%)	44 (41.5%)	1.281	0.258
Yes	164 (62.6%)	102 (65.4%)	62 (58.5%)		
Diarrhoea					
No	237 (90.5%)	143 (91.7%)	94 (88.7%)	0.653	0.419
Yes	25 (9.5%)	13 (8.3%)	12 (11.3%)		
Mouth ulcers					
No	252 (96.2%)	148 (94.9%)	104 (98.1%)	1.806	1.179
Yes	10 (3.8%)	8 (5.1%)	2 (1.9%)		
Alopecia					
No	111 (42.4%)	61 (39.1%)	50 (47.2%)	1.682	0.195
Yes	151 (57.6%)	95 (60.9%)	56 (52.8%)		
Peripheral neurotoxicity					
No	239 (91.2%)	141 (90.4%)	98 (92.5%)	0.337	0.561
Yes	23 (8.8%)	15 (9.6%)	8 (7.5%)		
Anaemia					
Grade 0	98 (37.4%)	56 (35.9%)	42 (39.6%)	1.654	0.437
Grade 1‐2	162 (61.8%)	98 (62.8%)	64 (60.4%)		
Grade 3‐4	2 (0.8%)	2 (1.3%)	0 (0.0%)		
Leukopenia					
Grade 0	77 (29.4%)	44 (28.2%)	33 (31.1%)	0.694	0.707
Grade 1‐2	106 (40.5%)	62 (39.7%)	44 (41.5%)		
Grade 3‐4	79 (30.1%)	50 (32.1%)	29 (27.4%)		
Neutropenia					
Grade 0	72 (27.5%)	42 (26.9%)	30 (28.3%)	1.767	0.413
Grade 1‐2	104 (39.7%)	58 (37.2%)	46 (43.4%)		
Grade 3‐4	86 (32.8%)	56 (35.9%)	30 (28.3%)		
Thrombocytopenia					
Grade 0	178 (67.9%)	100 (64.1%)	78 (73.6%)	2.605	0.272
Grade 1‐2	81 (30.9%)	54 (34.6%)	27 (25.5%)		
Grade 3‐4	3 (1.2%)	2 (1.3%)	1 (0.9%)		
Gastrointestinal reaction					
Grade 0	9 (3.4%)	6 (3.9%)	3 (2.8%)	2.285	0.319
Grade 1‐2	250 (95.4%)	147 (94.2%)	103 (97.2%)		
Grade 3‐4	3 (1.2%)	3 (1.9%)	0 (0.0%)		
Myelosuppression					
Grade 0	55 (21.0%)	34 (21.8%)	21 (19.8%)	0.337	0.845
Grade 1‐2	101 (38.5%)	58 (37.2%)	43 (40.6%)		
Grade 3‐4	106 (40.5%)	64 (41.0%)	42 (39.6%)		

### 3.6 Univariate and multivariate Cox regression survival analyses

**Figure 1:**
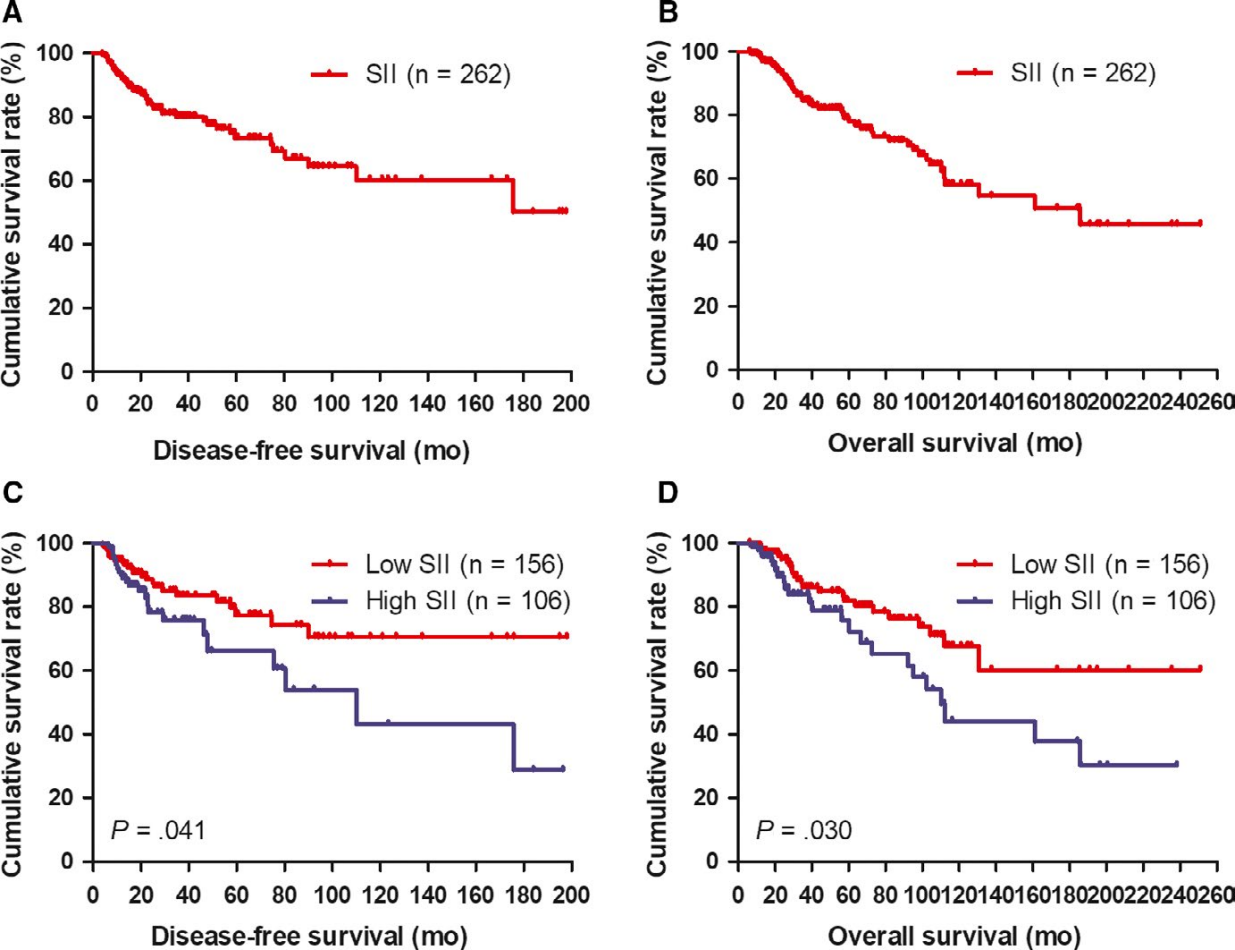
DFS and OS of patients with breast cancer. A, Kaplan‐Meier analysis of DFS of all patients with breast cancer. B, Kaplan‐Meier analysis of OS of all patients with breast cancer. C, Kaplan‐Meier analysis of DFS for the SII of all patients with breast cancer. D, Kaplan‐Meier analysis of OS for the SII of all patients with breast cancer. SII is a novel systemic immune‐inflammation index (SII = N×P/L), which is based on neutrophil (N), platelet (P) and lymphocyte (L) counts

The median DFS and OS of all enrolled patients were 36.85 months (range 4.00‐197.97 months) and 49.95 months (range 5.93‐250.97 months), respectively (Figure 1A, 1). The independent prognostic factors were performed univariate and multivariate Cox proportional hazards models based on time‐varying SII analysis. In univariate analysis, age, clinical T stage, clinical TNM stage, neoadjuvant chemotherapy times, tumour size, type of surgery, Miller and Payne grade, pathological T stage, pathological TNM stage, positive lymph nodes, core needle biopsy (ER status), post‐operative pathology IHC (molecular subtype, ER status, Ki‐67 status, AR status, CK5/6 status, EGFR status, TOP2A status), lymph vessel invasion, PLR, SII, post‐operative chemotherapy, post‐operative radiotherapy. Multivariate Cox regression analysis indicated that the factors related to DFS and OS to be tumour size, Miller and Payne grade, pathological T stage, pathological TNM stage, core needle biopsy (ER status), post‐operative pathology IHC (molecular subtype, ER status, Ki‐67 status, TOP2A status), lymph vessel invasion, PLR, SII. (Table 6).

**Table 6:** Univariate and multivariate Cox regression survival analyses of the SII for the prediction of DFS and OS in patients with breast cancer

Parameters	DFS				OS			
	Univariate analysis Hazard ratio (95%CI)	*P* value	Multivariate analysis Hazard ratio (95%CI)	*P* value	Univariate analysis Hazard ratio (95%CI)	*P* value	Multivariate analysis Hazard ratio (95%CI)	*P* value
Age (years)								
<48	1 (reference)	.042	1 (reference)	.655	1 (reference)	.001	1 (reference)	.101
≥48	4.071 (1.049‐15.799)		1.307 (0.405‐4.220)		2.125 (1.101‐9.862)		2.782 (0.820‐9.440)	
BMI								
<24.50	1 (reference)	.878			1 (reference)	.819		
≥24.50	1.037 (0.649‐1.659)				1.133 (0.388‐3.309)			
Menopause								
No	1 (reference)	.595			1 (reference)	.978		
Yes	1.140 (0.703‐1.848)				0.993 (0.583‐1.690)			
ABO blood type								
A	1 (reference)	.266			1 (reference)	.039	1 (reference)	.126
B	4.042 (0.892‐18.313)				3.694 (0.744‐18.346)		2.730 (1.011‐7.372)	
O	2.904 (0.689‐12.239)				7.527 (1.322‐42.873)		3.181 (1.118‐9.050)	
AB	3.683 (0.641‐21.178)				3.237 (1.040‐21.155)		2.601 (0.699‐9.676)	
Primary tumour site								
Upper outer quadrant	1 (reference)	.086			1 (reference)	.286		
Lower outer quadrant	0.427 (0.062‐2.917)				0.641 (0.078‐5.263)			
Lower inner quadrant	4.375 (1.710‐12.653)				0.627 (0.059‐5.948)			
Upper inner quadrant	0.736 (0.189‐2.861)				0.394 (0.065‐2.374)			
Central	2.218 (0.411‐11.954)				4.348 (0.556‐33.997)			
US‐LNM								
No	1 (reference)	.664			1 (reference)	.165		
Yes	1.114 (0.684‐1.815)				3.223 (0.618‐16.801)			
US‐BIRADS classification								
4	1 (reference)	.768			1 (reference)	.255		
5	0.902 (0.397‐2.049)				0.582 (0.239‐1.412)			
6	1.070 (0.473‐2.418)				0.813 (0.336‐1.969)			
Clinical stage								
Clinical T stage								
T1	1 (reference)	<.001	1 (reference)	.005	1 (reference)	.004	1 (reference)	.373
T2	1.141 (0.556‐2.341)		1.528 (0.493‐4.737)		1.532 (0.728‐3.222)		2.214 (0.662‐7.401)	
T3	1.187 (0.527‐2.673)		2.207 (0.559‐8.711)		1.435 (0.621‐3.315)		1.653 (0.408‐6.706)	
T4	0.858 (0.315‐2.338)		0.204 (0.049‐0.850)		1.602 (0.548‐4.683)		0.836 (0.153‐4.567)	
Clinical N stage								
N0	1 (reference)	.148			1 (reference)	.142		
N1	0.887 (0.519‐1.519)				1.222 (0.698‐2.140)			
N2	1.018 (0.566‐1.830)				1.263 (0.685‐2.327)			
N3	1.923 (0.923‐4.005)				2.347 (1.115‐4.944)			
Clinical TNM stage								
II	1 (reference)	<.001	1 (reference)	.364	1 (reference)	.020	1 (reference)	.794
III	1.682 (0.732‐3.862)		1.663 (0.555‐4.984)		1.342 (0.590‐3.054)		1.174 (0.352‐3.911)	
Neoadjuvant chemotherapy regimen								
AC/ACF	1 (reference)	.198			1 (reference)	.142		
CT/ACT	0.052 (0.003‐0.862)				0.307 (0.012‐7.971)			
AT	0.208 (0.034‐1.260)				2.488 (0.314‐19.730)			
TP	0.825 (0.145‐4.686)				5.524 (0.499‐61.157)			
Others	0.193 (0.014‐2.587)				0.098 (0.005‐2.063)			
Neoadjuvant chemotherapy times								
<6	1 (reference)	.022	1 (reference)	.604	1 (reference)	.003	1 (reference)	.028
≥6	4.257 (1.231‐14.720)		1.254 (0.534‐2.945)		2.030 (1.273‐3.235)		1.686 (1.058‐2.688)	
Response								
CR	1 (reference)	.973			1 (reference)	.207		
PR	1.046 (0.307‐3.558)				4.481 (0.976‐20.579)			
SD	1.144 (0.327‐4.008)				4.908 (1.054‐22.847)			
PD	1.239 (0.235‐6.532)				6.566 (1.003‐42.990)			
Tumour size								
≤2cm	1 (reference)	<.001	1 (reference)	.044	1 (reference)	<.001	1 (reference)	.002
>2 and < 5cm	0.848 (0.505‐1.424)		0.237 (0.077‐0.733)		0.911 (0.535‐1.551)		0.165 (0.057‐0.479)	
≥5cm	1.281 (0.610‐2.691)		0.338 (0.064‐1.776)		0.945 (0.423‐2.111)		0.089 (0.016‐0.495)	
Type of surgery								
Mastectomy	1 (reference)	.007	1 (reference)	.602	1 (reference)	.001	1 (reference)	.227
Breast‐conserving surgery	2.731 (1.128‐6.278)		1.323 (0.462‐3.794)		3.722 (1.416‐9.847)		1.965 (0.657‐5.874)	
Miller and Payne grade								
1	1 (reference)	.008	1 (reference)	<.001	1 (reference)	<.001	1 (reference)	<.001
2	0.442 (0.113‐1.726)		0.533 (0.267‐1.065)		0.297 (0.011‐7.895)		0.648 (0.127‐3.316)	
3	0.258 (0.067‐0.992)		0.298 (0.148‐0.600)		0.168 (0.004‐6.341)		0.623 (0.136‐2.845)	
4	0.017 (0.002‐0.151)		0.202 (0.076‐0.542)		0.000 (0.000‐0.000)		0.008 (0.001‐0.092)	
5	0.110 (0.009‐1.285)		0.146 (0.056‐0.378)		0.031 (0.000‐1.513)		0.190 (0.019‐1.074)	
Histologic type								
Ductal	1 (reference)	.092			1 (reference)	.253		
Lobular	2.309 (0.253‐21.070)				5.873 (0.618‐55.840)			
Others	3.523 (1.047‐11.861)				1.505 (0.392‐5.774)			
Histologic grade								
I	1 (reference)	.329			1 (reference)	.448		
II	1.006 (0.485‐2.088)				1.317 (0.600‐2.894)			
III	1.585 (0.662‐3.793)				1.759 (0.707‐4.374)			
Pathological TNM classification								
Pathological T stage								
Tis/T0	1 (reference)	<.001	1 (reference)	.004	1 (reference)	<.005	1 (reference)	.001
T1	3.458 (1.881‐7.919)		2.108 (0.709‐5.933)		3.785 (1.745‐6.360)		2.213 (0.812‐7.844)	
T2	1.741 (0.669‐3.382)		1.574 (0.354‐4.203)		1.892 (1.932‐5.078)		1.481 (0.993‐7.638)	
T3	1.364 (0.259‐4.152)		1.577 (0.795‐5.046)		1.890 (1.609‐5.104)		1.085 (0.940‐4.104)	
T4	1.226 (0.444‐3.467)		1.596 (0.864‐4.061)		2.027 (1.092‐5.873)		1.334 (1.179‐5.151)	
Pathological N stage								
N0	1 (reference)	.674			1 (reference)	.264		
N1	1.666 (0.465‐5.968)				1.760 (0.439‐7.058)			
N2	1.484 (0.428‐5.144)				2.846 (0.763‐10.612)			
N3	1.918 (0.606‐6.072)				3.329 (0.963‐11.512)			
Pathological TNM stage								
Tis/T0	1 (reference)	.004	1 (reference)	<.001	1 (reference)	<.001	1 (reference)	<.001
I	1.510 (1.028‐4.285)		2.294 (0.976‐5.391)		3.023 (1.213‐7.532)		2.474 (1.015‐6.031)	
II	1.017 (0.535‐4.736)		1.559 (0.660‐3.686)		1.084 (0.428‐2.747)		1.091 (0.449‐2.654)	
III	1.016 (0.445‐3.585)		2.436 (1.118‐5.308)		1.120 (0.412‐3.040)		3.943 (1.756‐8.856)	
Total lymph nodes								
<21	1 (reference)	.074			1 (reference)	.625		
≥21	0.380 (0.132‐1.099)				0.669 (0.133‐3.367)			
Positive lymph nodes								
0	1 (reference)	.001	1 (reference)	.741	1 (reference)	.004	1 (reference)	.468
<6	1.347 (0.956‐4.208)		0.837 (0.231‐3.034)		1.967 (1.113‐4.079)		0.587 (0.145‐2.378)	
≥6	1.405 (0.925‐5.094)		1.266 (0.421‐3.807)		1.278 (1.080‐6.502)		1.190 (0.369‐3.843)	
Core needle biopsy								
Molecular subtype								
Luminal A	1 (reference)	.196			1 (reference)	.001	1 (reference)	.003
Luminal B HER2+	0.298 (0.001‐1.854)				1.234 (0.654‐4.150)		1.461 (0.857‐4.691)	
Luminal B HER2‐	3.174 (0.146‐9.040)				1.318 (0.040‐3.033)		1.915 (0.102‐8.236)	
HER2 enriched	1.889 (0.008‐8.686)				1.678 (0.884‐3.324)		1.785 (0.997‐6.555)	
Triple negative	2.433 (0.631‐4.473)				1.548 (0.372‐4.718)		1.623 (0.761‐3.917)	
ER status								
Negative	1 (reference)	.001	1 (reference)	.030	1 (reference)	<.001	1 (reference)	.048
Positive	13.911 (2.793‐69.277)		4.020 (1.143‐14.142)		1.514 (1.300‐11.659)		4.737 (1.011‐22.186)	
PR status								
Negative	1 (reference)	.047	1 (reference)	.632	1 (reference)	.08	1 (reference)	.399
Positive	4.902 (1.018‐23.600)		1.329 (0.415‐4.249)		6.796 (0.795‐58.111)		1.876 (0.434‐8.105)	
HER2 status								
Negative (0‐‐++)	1 (reference)	.436			1 (reference)	.822		
Positive (+++)	1.294 (0.676‐2.476)				1.080 (0.552‐2.114)			
Ki‐67 status								
Negative (≤14%)	1 (reference)	.087			1 (reference)	.002	1 (reference)	.005
Positive (>14%)	2.685 (0.868‐8.309)				2.147 (1.327‐3.473)		1.976 (1.226‐3.188)	
Post‐operative pathology (IHC)								
Molecular subtype								
Luminal A	1 (reference)	.005	1 (reference)	.005	1 (reference)	.001	1 (reference)	.002
Luminal B HER2+	0.003 (0.000‐0.143)		0.006 (0.000‐0.110)		0.000 (0.000‐0.010)		0.001 (0.000‐0.035)	
Luminal B HER2‐	0.002 (0.000‐0.059)		0.041 (0.004‐0.404)		0.007 (0.000‐0.266)		0.046 (0.004‐0.476)	
HER2 enriched	0.013 (0.000‐0.579)		0.005 (0.000‐0.277)		0.000 (0.000‐0.026)		0.000 (0.000‐0.060)	
Triple negative	0.005 (0.000‐0.430)		0.010 (0.000‐0.285)		0.000 (0.000‐0.015)		0.018 (0.000‐0.742)	
ER status								
Negative	1 (reference)	.014	1 (reference)	.039	1 (reference)	.002	1 (reference)	.036
Positive	1.097 (0.731‐1.645)		1.148 (0.787‐1.676)		1.180 (0.771‐1.807)		1.334 (0.910‐1.956)	
PR status								
Negative	1 (reference)	.084			1 (reference)	.01	1 (reference)	.012
Positive	3.830 (0.833‐17.600)				1.787 (1.149‐2.778)		1.742 (1.131‐2.682)	
HER2 status								
Negative (0‐‐++)	1 (reference)	.719			1 (reference)	.001	1 (reference)	.015
Positive (+++)	1.917 (0.055‐66.366)				1.432 (0.968‐4.094)		1.759 (1.118‐2.767)	
Ki‐67 status								
Negative (≤14%)	1 (reference)	.004	1 (reference)	.004	1 (reference)	<.001	1 (reference)	.009
Positive (>14%)	3.875 (1.544‐9.724)		1.793 (1.200‐2.678)		2.707 (1.811‐7.339)		2.658 (0.927‐7.622)	
AR status								
Negative	1 (reference)	.001	1 (reference)	.124	1 (reference)	.005	1 (reference)	.564
Positive	0.109 (0.028‐0.427)		0.453 (0.165‐1.242)		0.085 (0.015‐0.473)		0.751 (0.284‐1.986)	
CK5/6 status								
Negative	1 (reference)	.004	1 (reference)	.149	1 (reference)	.011	1 (reference)	.279
Positive	0.036 (0.004‐0.347)		0.331 (0.074‐1.485)		0.026 (0.002‐0.432)		0.410 (0.082‐2.059)	
E‐cad status								
Negative	1 (reference)	.354			1 (reference)	.961		
Positive	1.310 (0.740‐2.322)				1.040 (0.218‐4.950)			
EGFR status								
Negative	1 (reference)	.008	1 (reference)	.296	1 (reference)	.024	1 (reference)	.266
Positive	0.089 (0.015‐0.532)		0.565 (0.194‐1.646)		0.087 (0.011‐0.723)		0.499 (0.147‐1.697)	
P53 status								
Negative	1 (reference)	.988			1 (reference)	.884		
Positive	1.004 (0.593‐1.700)				1.041 (0.609‐1.779)			
TOP2A status								
Negative	1 (reference)	.001	1 (reference)	.037	1 (reference)	<.001	1 (reference)	.001
Positive	0.126 (0.035‐0.450)		0.359 (0.137‐0.939)		0.027 (0.005‐0.143)		0.163 (0.056‐0.476)	
Lymph vessel invasion								
Negative	1 (reference)	.001	1 (reference)	.024	1 (reference)	.004	1 (reference)	.033
Positive	1.556 (0.812‐2.983)		3.056 (1.158‐8.061)		1.172 (0.592‐2.319)		1.587 (0.577‐4.366)	
Neural invasion								
Negative	1 (reference)	.816			1 (reference)	.513		
Positive	1.053 (0.684‐1.621)				1.149 (0.757‐1.744)			
CA125								
<27.73	1 (reference)	.149			1 (reference)	<.001	1 (reference)	.16
≥27.73	0.383 (0.104‐1.411)				0.027 (0.004‐0.181)		0.428 (0.131‐1.399)	
CA153								
<21.86	1 (reference)	.368			1 (reference)	.261		
≥21.86	0.576 (0.173‐1.916)				0.383 (0.072‐2.044)			
CEA								
<3.52	1 (reference)	.253			1 (reference)	.061		
≥3.52	1.817 (0.652‐5.063)				4.312 (0.932‐19.943)			
White blood cell (W)								
<6.00	1 (reference)	.333			1 (reference)	.548		
≥6.00	1.369 (0.724‐2.587)				1.222 (0.635‐2.350)			
Red blood cell (R)								
<4.34	1 (reference)	.989			1 (reference)	.584		
≥4.34	1.006 (0.467‐2.164)				0.679 (0.170‐2.716)			
Haemoglobin (Hb)								
<128.00	1 (reference)	.778			1 (reference)	.679		
≥128.00	1.095 (0.582‐2.061)				1.154 (0.585‐2.276)			
Neutrophil (N)								
<3.83	1 (reference)	.253			1 (reference)	.446		
≥3.83	1.308 (0.826‐2.070)				1.192 (0.759‐1.871)			
Lymphocyte (L)								
<1.67	1 (reference)	.141			1 (reference)	.246		
≥1.67	0.298 (0.059‐1.496)				0.336 (0.053‐2.123)			
Monocyte (M)								
<0.34	1 (reference)	.496			1 (reference)	.76		
≥0.34	1.570 (0.428‐5.756)				1.237 (0.315‐4.863)			
Platelet (P)								
<244.00	1 (reference)	.724			1 (reference)	.241		
≥244.00	0.822 (0.278‐2.435)				0.407 (0.090‐1.832)			
NLR								
<2.50	1 (reference)	.681			1 (reference)	.69		
≥2.50	1.164 (0.564‐2.403)				1.184 (0.516‐2.720)			
MLR								
<0.22	1 (reference)	.47			1 (reference)	.557		
≥0.22	1.447 (0.530‐3.948)				1.167 (0.697‐1.953)			
PLR								
<160.00	1 (reference)	.004	1 (reference)	.030	1 (reference)	<.001	1 (reference)	.018
≥160.00	1.319 (0.783‐2.221)		2.450 (0.928‐6.471)		1.139 (0.664‐1.955)		3.736 (1.251‐11.153)	
SII								
<602	1 (reference)	.003	1 (reference)	.006	1 (reference)	<.001	1 (reference)	.005
≥602	1.582 (1.178‐4.158)		1.075 (0.718‐1.610)		1.472 (0.387‐4.681)		1.051 (0.707‐1.564)	
Post‐operative chemotherapy								
No	1 (reference)	.003	1 (reference)	.006	1 (reference)	.033	1 (reference)	.148
Yes	9.618 (2.117‐43.704)		4.500 (1.552‐13.050)		5.265 (1.146‐24.188)		2.144 (0.762‐6.030)	
Post‐operative radiotherapy								
No	1 (reference)	<.001	1 (reference)	.618	1 (reference)	<.001	1 (reference)	.033
Yes	0.097 (0.028‐0.333)		0.783 (0.300‐2.047)		0.021 (0.004‐0.112)		0.322 (0.113‐0.913)	
Post‐operative endocrine therapy								
No	1 (reference)	.469			1 (reference)	.029	1 (reference)	.431
Yes	0.660 (0.214‐2.034)				0.248 (0.071‐0.865)		0.674 (0.253‐1.797)	
Post‐operative targeted therapy								
No	1 (reference)	.294			1 (reference)	.867		
Yes	1.915 (1.023‐3.587)				1.163 (0.198‐6.812)			

Patients with low SII had significantly lower risks of disease progression compared with those patients with high SII. Moreover, patients with low SII value were related to prolonged DFS and OS by univariate analysis (*P* = .003, hazard ratio (HR): 1.582, 95% confidence interval (CI): 1.178‐4.518 and *P* < .001, HR: 1.472, 95% CI: 0.387‐4.681, respectively). And low SII was also related to prolonged DFS and OS (*P* = .006, HR: 1.075, 95% CI: 0.718‐1.610 and *P* = .005, HR: 1.051, 95% CI: 0.707‐1.564, respectively; Table 6). Nevertheless, the mean DFS and OS for all enrolled cases with low SII were 40.76 months (range from 4.00‐197.97 months) and 53.68 months (range from 5.93‐250.97 months), respectively, and the mean DFS and OS for all patients with high SII were 31.11 months (range from 6.27‐196.40 months) and 44.47 months (range from 7.30‐238.27 months), respectively. The mean DFS and OS time in patients with low SII were significantly longer than that in those patients with high SII by using log‐rank methods (*χ* ^2^ = 4.184, *P* = .041 and *χ* ^2^ = 4.692, *P* = .030, respectively; Figure 1C, 1).

### 3.7 Survival rate and prognostic significance of SII

**Figure 2:**
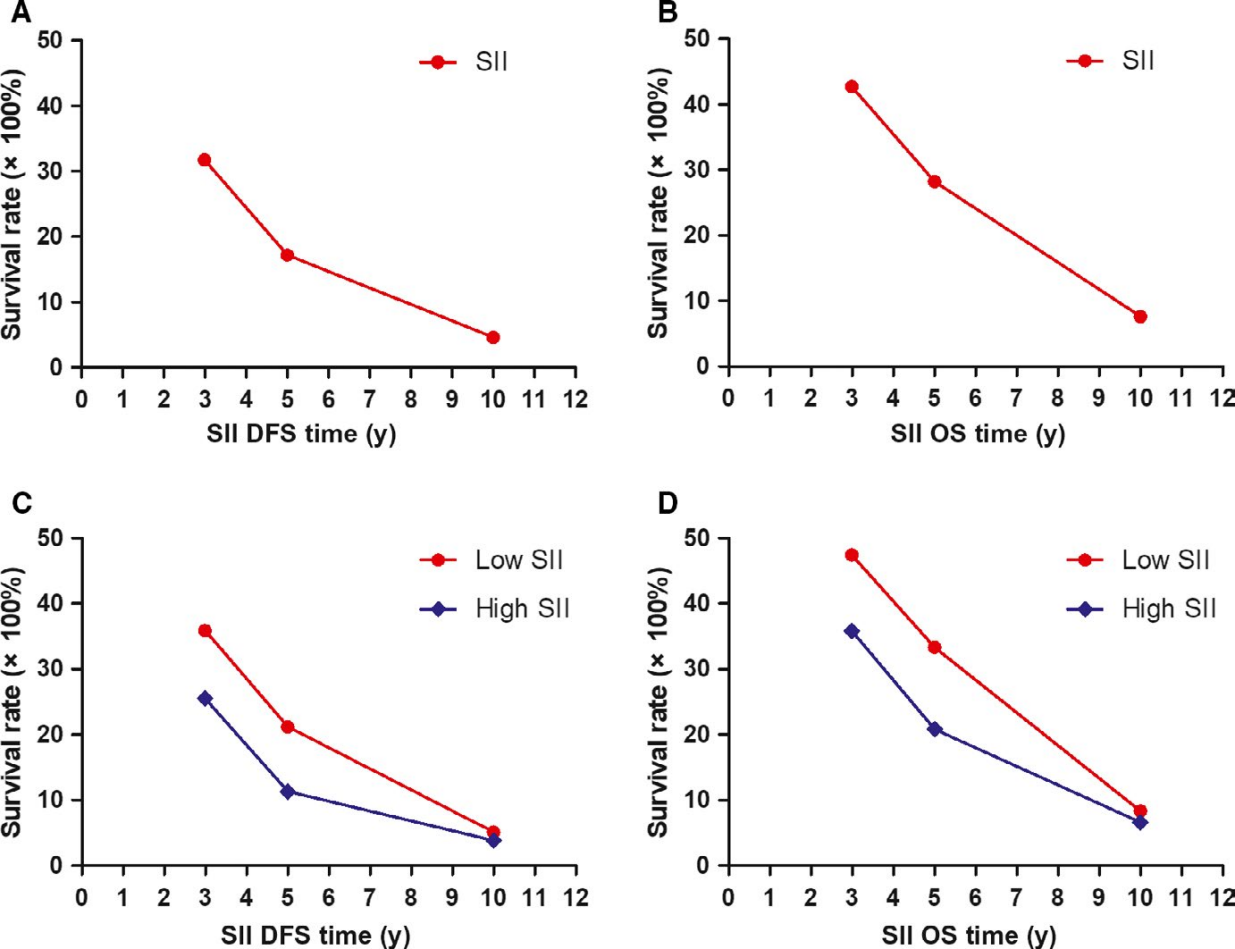
The 3‐, 5‐ and 10‐year rates of DFS and OS in patients with breast cancer. A, The 3‐, 5‐ and 10‐year rates of DFS in all patients with breast cancer. B, The 3‐, 5‐ and 10‐year rates of OS in all patients with breast cancer. C, The 3‐, 5‐ and 10‐year rates of DFS in all patients by SII with breast cancer. D, The 3‐, 5‐ and 10‐year rates of OS in all patients by SII with breast cancer

The DFS rates at 3‐, 5‐ and 10‐year were 31.7% (83/262), 17.2% (45/262), 4.6% (12/262); the OS rates at 3‐, 5‐ and 10‐year were 42.7% (112/262), 28.2% (74/262), 7.6% (20/262), respectively. Moreover, the DFS rates at 3‐, 5‐ and 10‐year in low SII were 35.9% (56/156), 21.2% (33/156), 5.1% (8/156), and the OS rates at 3‐, 5‐ and 10‐year in low SII were 47.4% (74/156), 33.3% (52/156), 8.3% (13/156), respectively. The DFS rates at 3‐, 5‐ and 10‐year in high SII were 25.5% (27/106), 11.3% (12/106), 3.8% (4/106), and the OS rates at 3‐, 5‐ and 10‐year in high SII were 35.8% (38/106), 20.8% (22/106), 6.6% (7/106), respectively. The results also indicated that the incidence of DFS and OS in patients with low SII was higher than that in patients with high SII in 3‐, 5‐ and 10‐year rates. Although there was a trend towards significance for the association between low SII and high SII at 3‐, 5‐ and 10‐year rates, there were no significant differences between the two groups. (Table 7, Figure 2A‐D).

**Table 7:** 3‐, 5‐ and 10‐year DFS and OS rates of patients with breast cancer

Parameters	Cases (n)	DFS			OS		
		3‐year (%)	5‐year (%)	10‐year (%)	3‐year (%)	5‐year (%)	10‐year (%)
Total	262	83/262 (31.7%)	45/262 (17.2%)	12/262 (4.6%)	112/262 (42.7%)	74/262 (28.2%)	20/262 (7.6%)
Low SII	156	56/156 (35.9%)	33/156 (21.2%)	8/156 (5.1%)	74/156 (47.4%)	52/156 (33.3%)	13/156 (8.3%)
High SII	106	27/106 (25.5%)	12/106 (11.3%)	4/106 (3.8%)	38/106 (35.8%)	22/106 (20.8%)	7/106 (6.6%)
*χ* ^2^		2.542	3.601	0.199	2.769	0.138	0.21
*P* value		.111	.058	.656	.096	.71	.647

### 3.8 Association of molecular subtypes by post‐operative pathology IHC and SII in patients with breast carcinoma

**Figure 3:**
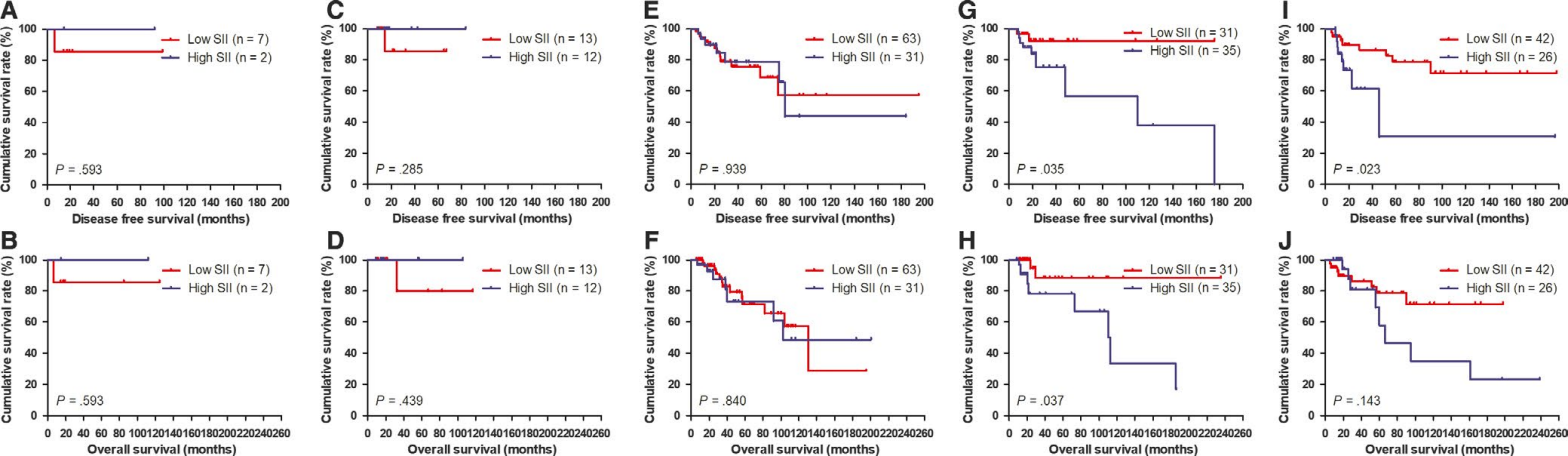
DFS and OS of patients for the SII by molecular subtypes with breast cancer. A, Kaplan‐Meier analysis of DFS of patients by Luminal A subtype with breast cancer. B, Kaplan‐Meier analysis of OS of patients by Luminal A subtype with breast cancer. C, Kaplan‐Meier analysis of DFS of patients by Luminal B HER2‐positive subtype with breast cancer. D, Kaplan‐Meier analysis of OS of patients by Luminal B HER2‐positive subtype with breast cancer. E, Kaplan‐Meier analysis of DFS of patients by Luminal B HER2‐negative subtype with breast cancer. F, Kaplan‐Meier analysis of OS of patients by Luminal B HER2‐negative subtype with breast cancer. G, Kaplan‐Meier analysis of DFS of patients by HER2‐enriched subtype with breast cancer. H, Kaplan‐Meier analysis of OS of patients by HER2‐enriched subtype with breast cancer. I, Kaplan‐Meier analysis of DFS of patients by Triple negative subtype with breast cancer. J, Kaplan‐Meier analysis of OS of patients by Triple negative subtype with breast cancer

The results showed that molecular subtypes by post‐operative pathology IHC were the important prognostic factor (Table 6). In order to go deep into evaluating the prognostic value of SII, the SII was analysed by the molecular subtypes. And the SII with different molecular subtypes was analysed by the log‐rank test (Figure 3). For all enrolled patients with breast carcinoma, the results indicated that the DFS and OS time in patients with low SII were significantly longer than that in those patients with high SII in HER2‐enriched subtype (*χ* ^2^ = 4.448, *P* = .035 and *χ* ^2^ = 4.371, *P* = .037, respectively; Figure 3G, H). Meanwhile, the DFS and OS time in patients with low SII were significantly longer than that in those patients with high SII in triple negative subtype (*χ* ^2^ = 5.146, *P* = .023 and *χ* ^2^ = 2.150, *P* = .143, respectively; Figure 3I, J).

### 3.9 Correlation between Miller and Payne grade (MPG) and SII in breast cancer patients

**Figure 4:**
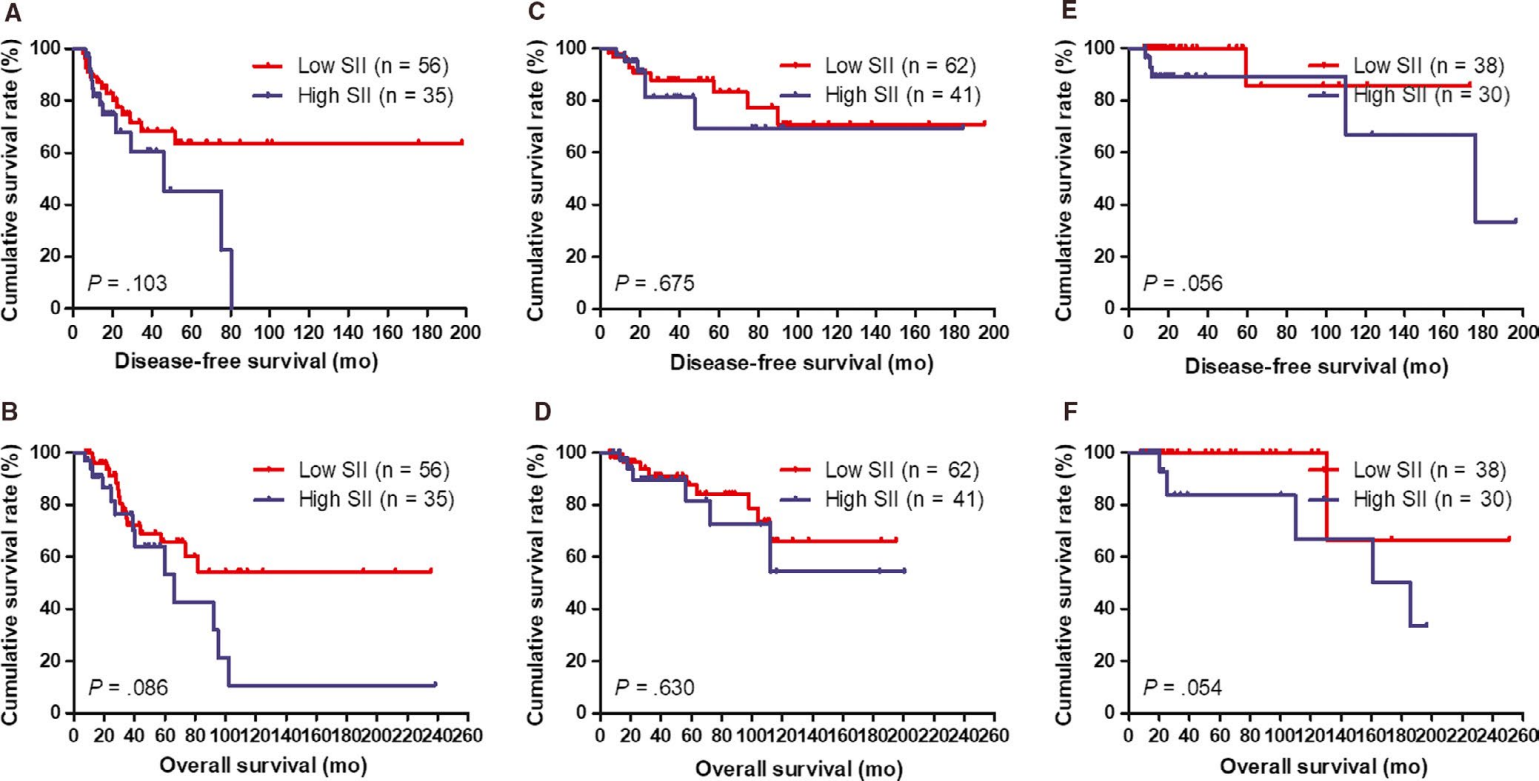
DFS and OS of patients for the SII by Miller and Payne grade (MPG) with breast cancer. A, Kaplan‐Meier analysis of DFS of patients by MPG‐A group with breast cancer. B, Kaplan‐Meier analysis of OS of patients by MPG‐A group with breast cancer. C, Kaplan‐Meier analysis of DFS of patients by MPG‐B group with breast cancer. D, Kaplan‐Meier analysis of OS of patients by MPG‐B group with breast cancer. E, Kaplan‐Meier analysis of DFS of patients by MPG‐C group with breast cancer. F, Kaplan‐Meier analysis of OS of patients by MPG‐C group with breast cancer

According to univariate and multivariate analyses, the MPG was the significant prognostic factor (Table 3). We also analysed the SII by MPG. We defined the patients with MPG grade 1 and 2 as MPG‐A group, the patients with MPG grade 3 as MPG‐B group and patients with MPG grade 4 and 5 as MPG‐C group, respectively. By using the log‐rank test, the mean DFS and OS time for patients with low SII were longer than in those with high SII in MPG‐A group (*χ* ^2^ = 2.657, *P* = .103 and *χ* ^2^ = 2.953, *P* = .086, respectively; Figure 4A, 4). The mean DFS and OS time in patients with low SII were longer than that in those patients with high SII in MPG‐B group (*χ* ^2^ = 0.176, *P* = .675 and *χ* ^2^ = 0.232, *P* = .630, respectively; Figure 4C, 4). The mean DFS and OS time in patients with low SII were longer than that in those patients with high SII in MPG‐C group (*χ* ^2^ = 3.652, *P* = .056 and *χ* ^2^ = 3.709, *P* = .054, respectively; Figure 4E, 4).

### 3.10 Association of ER status and SII in patients with breast carcinoma

**Figure 5:**
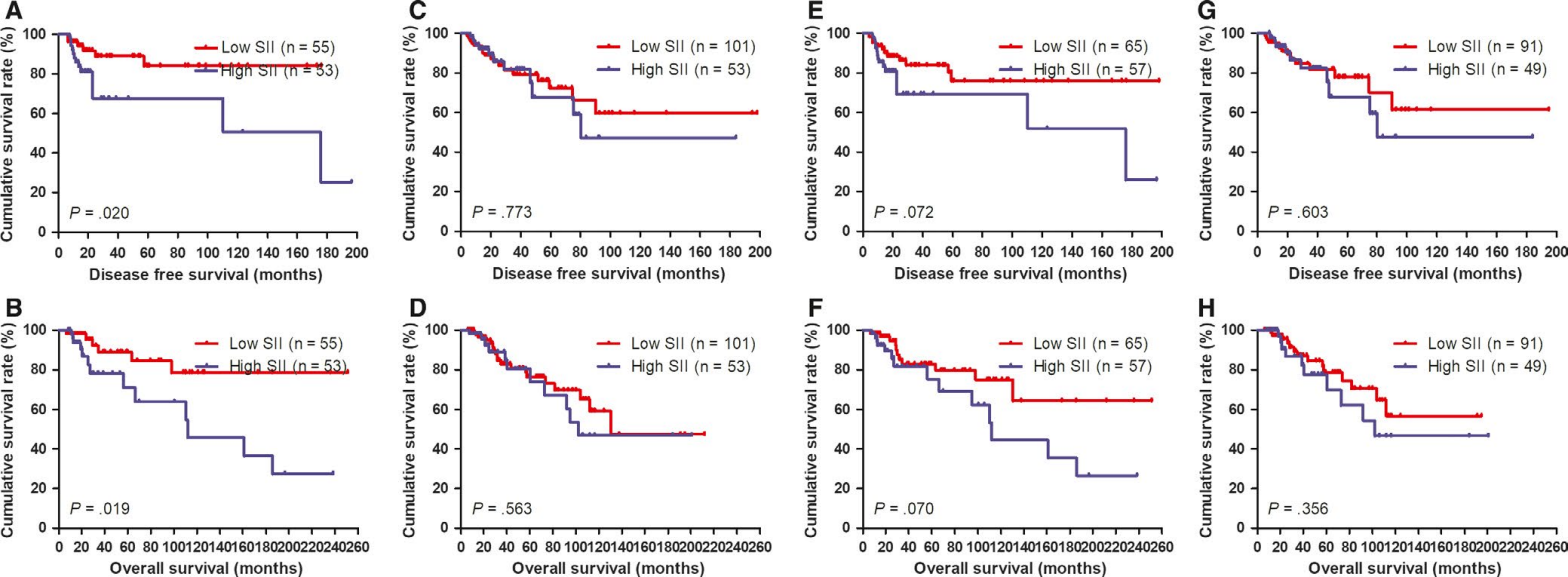
DFS and OS of patients for the SII by ER status with breast cancer. A, Kaplan‐Meier analysis of DFS of patients by ER negative with breast cancer in core needle biopsy. B, Kaplan‐Meier analysis of OS of patients by ER negative with breast cancer in core needle biopsy. C, Kaplan‐Meier analysis of DFS of patients by ER positive with breast cancer in core needle biopsy. D, Kaplan‐Meier analysis of OS of patients by ER positive with breast cancer in core needle biopsy. E, Kaplan‐Meier analysis of DFS of patients by ER negative with breast cancer in post‐operative pathology IHC. F, Kaplan‐Meier analysis of OS of patients by ER negative with breast cancer in post‐operative pathology IHC. G, Kaplan‐Meier analysis of DFS of patients by ER positive with breast cancer in post‐operative pathology IHC. H, Kaplan‐Meier analysis of OS of patients by ER positive with breast cancer in post‐operative pathology IHC

The results indicated that ER status in both core needle biopsy and post‐operative pathology IHC was the significant prognostic factor. Hence, for the sake of further to study the prognostic efficiency of SII, the SII was analysed by ER status. In core needle biopsy, the mean DFS and OS time in patients with low SII by the log‐rank test were longer than in those patients with high SII in ER negative (*χ* ^2^ = 5.401, *P* = .020 and *χ* ^2^ = 5.476, *P* = .019, respectively; Figure 5A, 5). And the mean DFS and OS time in patients with low SII by the log‐rank test were longer than in those patients with high SII in ER positive (*χ* ^2^ = 0.083, *P* = .773 and *χ* ^2^ = 0.335, *P* = .563, respectively; Figure 5C, 5). In post‐operative pathology IHC, the mean DFS and OS time in patients with low SII by the log‐rank test were longer than in those patients with high SII in ER negative (*χ* ^2^ = 3.233, *P* = .072 and *χ* ^2^ = 3.289, *P* = .070, respectively; Figure 5E, 5). And the mean DFS and OS time in patients with low SII by the log‐rank test were longer than in those patients with high SII in ER positive (*χ* ^2^ = 0.271, *P* = .603 and *χ* ^2^ = 0.852, *P* = .356, respectively; Figure 5G, H).

### 3.11 Correlation between Ki‐67 status and SII in breast cancer patients

**Figure 6:**
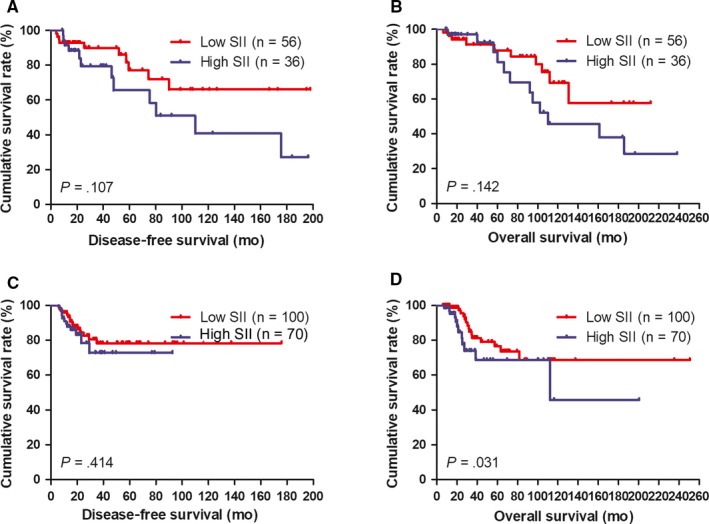
DFS and OS of patients for the SII by Ki‐67 status with breast cancer. A, Kaplan‐Meier analysis of DFS of patients by Ki‐67 negative with breast cancer. B, Kaplan‐Meier analysis of OS of patients by Ki‐67 negative with breast cancer. C, Kaplan‐Meier analysis of DFS of patients by Ki‐67 positive with breast cancer. D, Kaplan‐Meier analysis of OS of patients by Ki‐67 positive with breast cancer

According to univariate and multivariate analyses, Ki‐67 status in post‐operative pathology IHC was the significant prognostic factor. The results showed that the mean DFS and OS time in patients with low SII by the log‐rank test were longer than in those patients with high SII in Ki‐67 negative (*χ* ^2^ = 2.601, *P* = .107 and *χ* ^2^ = 2.161, *P* = .142, respectively; Figure 6A, 6). And the results showed that the mean DFS and OS time in patients with low SII by the log‐rank test were longer than in those patients with high SII in Ki‐67 positive (*χ* ^2^ = 0.667, *P* = .414 and *χ* ^2^ = 4.667, *P* = .031, respectively; Figure 6C, 6). Patients with breast cancer who had lower SII and Ki‐67 negative would have survived longer than those patients with high SII and Ki‐67 positive.

### 3.12 Association of lymph vessel invasion and SII in breast cancer patients

**Figure 7:**
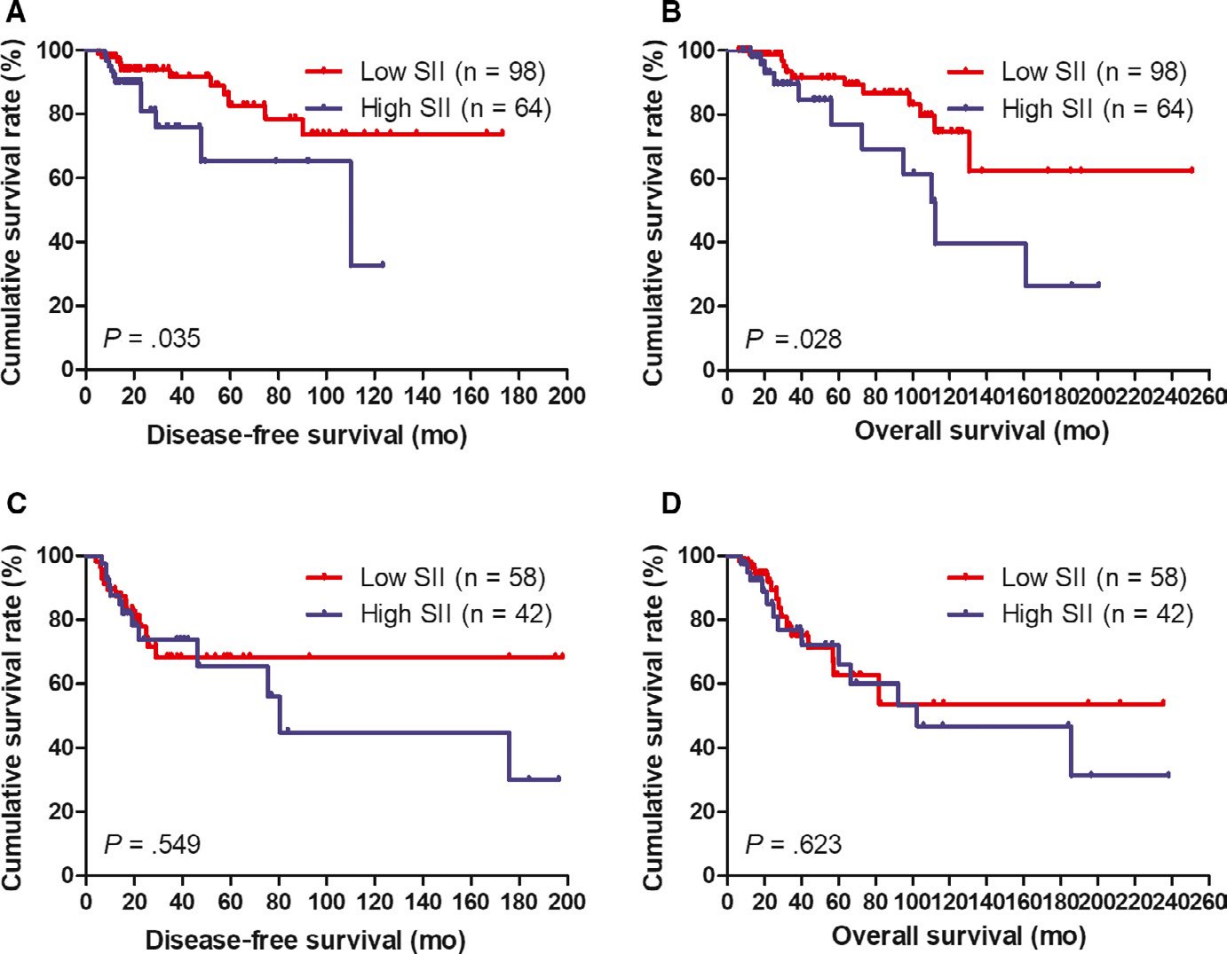
DFS and OS of patients for the SII by lymph vessel invasion status with breast cancer. A, Kaplan‐Meier analysis of DFS of patients by lymph vessel invasion negative with breast cancer. B, Kaplan‐Meier analysis of OS of patients by lymph vessel invasion negative with breast cancer. C, Kaplan‐Meier analysis of DFS of patients by lymph vessel invasion positive with breast cancer. D, Kaplan‐Meier analysis of OS of patients by lymph vessel invasion positive with breast cancer

The results also proved that lymph vessel invasion was the significant prognostic factor by univariate and multivariate analyses (Table 6). The results showed that the mean DFS and OS time in patients with low SII by the log‐rank test were longer than in those patients with high SII in patients without lymph vessel invasion (*χ* ^2^ = 4.438, *P* = .035 and *χ* ^2^ = 4.817, *P* = .028, respectively; Figure 7A, 7). And the results also indicated that the mean DFS and OS time in patients with low SII by the log‐rank test were longer than in those patients with high SII in patients with lymph vessel invasion (*χ* ^2^ = 0.359, *P* = .549 and *χ* ^2^ = 0.241, *P* = .623, respectively; Figure 7C, 7).

## 4 DISCUSSION

Breast carcinoma is the most common diagnostic cancer and with more than 2.1 million newly diagnosed female breast cancer cases in 2018, accounting for almost one in four cancer cases among women.^36^ The surgery, radiotherapy and endocrine therapy are used to the treatment of early breast cancer; however, the neoadjuvant treatment which can turn inoperable tumours into operable tumours or reduce tumour stage for more frequent conservative breast surgery.^37^ Meanwhile, NACT allows rapid assessment of drug efficacy, and the clinical outcomes and quality of life for patients with breast cancer have greatly improved, especially in TNBC.^38^ Hence, it is very important to look for precise predictors to choose the best treatment options and improve clinical outcomes for breast cancer patients.

Peripheral venous blood analysis can proclaim the condition of the whole immune system. Some studies have indicated the association between the systemic inflammation and malignant tumours.^39^, ^40^, ^41^, ^42^ The neutrophils, monocytes, platelets and lymphocytes are associated with the prognosis of different kinds of cancer. Neutrophils are a maker of inflammatory and immune response and influence tumour development, progression and metastasis via restraining inflammatory factors, such as neutrophil elastase, matrix metalloproteinase‐9 (MMP‐9), nuclear factor‐κB (NF‐kB), vascular endothelial growth factor (VEGF) and interleukin‐8 (IL‐8) have been clarified.^43^, ^44^, ^45^, ^46^, ^47^ Monocytes play an important role in tumour angiogenesis, inflammatory response and metastases and can create an environment of chronic oxidative stress, such as oncostain‐M, VEGF has been proved.^48^, ^49^, ^50^ Platelets have been found to contribute to an indicator of tumour activity, and relate to growth, invasion and metastasis of the primary tumour; and platelet release promotes cancer cell proliferation mainly through VEGF‐integrin cooperative signalling.^51^, ^52^, ^53^ However, lymphocytes are involved in the prevention of tumour proliferation, and the lymphocyte response is an important component of anticancer immunity and immune surveillance, as well as lymphocytes are implicated in the killing of cancer cells and increased chemotherapy responsiveness, and correlates with better survival in cancer patients.^54^, ^55^, ^56^, ^57^

Numerous studies showed that inflammatory markers and immune‐based prognostic indexes, such as NLR, d‐NLR, MLR/LMR and PLR, were considered to have prognostic value for breast cancer.^58^, ^59^, ^60^, ^61^, ^62^, ^63^, ^64^, ^65^, ^66^ The GEICAM 9906 trial indicated that patients with high NLR were associated with shorter DFS in HER2‐enriched subtype (*P* = .03), and high NLR was identified as an independent predictor by univariate and multivariate analysis.^58^ A recent meta‐analysis showed that the NLR was related to poor survival time and regarded as a predictive and prognostic factor with 2267 Chinese and Caucasian patients with breast cancer.^59^ A retrospective study indicated that high LMR value and low NLR value were related to a lower risk of relapse (*P* = .048 and *P* = .015, respectively) and patients with low NLR would survival longer than those patients with high NLR (*P* = .024).^60^ A study of 239 Japan patients with breast cancer proved that LMR may be a useful prognostic marker in DFS (*P* = .005) and could predict the progression and chemosensitivity in patients with breast cancer treated with pre‐operative chemotherapy.^61^ Another retrospective study proclaimed that LMR was significantly related to a poor prognosis for TNBC subtype of 1570 Chinese breast cancer patients (*P* = .041).^62^

Meanwhile, a meta‐analysis revealed that low LMR was significantly related to poor OS (*P* = .009) and DFS (*P* < .001) with 5667 individuals, and the LMR might be a predictive factor of poor prognosis for patients with breast cancer.^63^ In the study by Asano and colleagues, patients with low PLR treated with neoadjuvant chemotherapy exhibited higher rates of pCR, DFS, and OS, and PLR was a predictive and prognostic biomarker for patients with triple negative breast cancer.^64^ The Irish Clinical Oncology Group study (ICORG 16‐20) also found that high PLR was independently associated with poor response to neoadjuvant chemotherapy for patients with breast cancer.^65^ Another study by Cuello‐López J et al indicated that breast cancer patients with low PLR treated with NACT achieved higher complete pathological response and had been proposed as predictive factors of response to NACT.^66^ Although these inflammatory markers were associated with poor prognosis in patients with breast cancer, the exact results and potential mechanisms were still undefined. The NLR, LMR/MLR and PLR are based on two inflammatory cells and may predict the prognosis of malignant tumours.

However, SII based on three inflammatory cells, including neutrophil, platelet and lymphocyte and can fully reflect the balance between host immune and inflammatory status. SII has been investigated in some malignancies, such as gastric cancer, prostate cancer, lung cancer and pancreatic cancer, and SII has been proved to be an independent predictor of malignant tumours.^67^, ^68^, ^69^, ^70^ In our study, we confirmed that the SII was the significant prognostic factor by univariate and multivariate analyses and could predict survival in breast cancer patients receiving NACT.

In our study, the clinicopathologic and demographic characteristics of the 262 patients with breast cancer were enrolled and analysed. The optimal cut‐off value of the SII was 602 × 10^9^/L by ROC analysis, and this value was used to the data analysis. The results indicated that the low SII was significantly correlated with menopause and US‐LNM. The blood parameters were determined by its median value, and the low SII was significantly correlated with ALB, CRP, CA125, W, R, Hb, N, P, NLR, MLR and PLR. Meanwhile, low SII was significantly related to core needle biopsy ER status, post‐operative pathology IHC HER2 status. Moreover, the common toxicities were haematological and gastrointestinal reaction after neoadjuvant chemotherapy.

According to univariate and multivariate Cox regression analyses, tumour size, Miller and Payne grade, pathological T stage, pathological TNM stage, core needle biopsy (ER status), post‐operative pathology IHC (molecular subtype, ER status, Ki‐67 status, TOP2A status), lymph vessel invasion, PLR, and SII were important factors of prognosis. The results determined that SII had prognostic significance by using the cut‐off value of 602 × 10^9^/L for DFS and OS time, and patients with low SII had survival longer than those patients with high SII. Meanwhile, the results also indicated that patients with low SII have longer 3‐, 5‐ and 10‐year rates of DFS and OS time. Moreover, we also found that molecular subtypes were the significant prognostic factors, and patients with low SII would have better survival outcome than those with high SII in triple negative subtype.

The proliferation activity of tumours is usually determined with immunohistochemical detection of the cell‐cycle‐specific antigen Ki‐67. Some studies have proved that Ki‐67 expression is a useful prognostic factor in breast cancer.^71^, ^72^ Our results also indicated that Ki‐67 status in post‐operative pathology IHC was the significant prognostic factor, and patients with low SII would have survive longer than those with high SII by Ki‐67 status. And further to study the ER status, patients with low SII would survival longer than those patients with high SII by ER negative. The MPG was used to evaluate the histologic response, MPG was the significant prognostic factor, and patients with high MPG grade would survival longer than those with low MPG grade on DFS and OS time. Lymphatic vessel is confided to play a passive role in tumour metastasis, such as the provision of a niche for cancer stem cells and the modulation of antitumour immune responses.^73^ And tumour angiogenesis and its indicator blood vessel density are closely related to the prognosis in breast cancer.^74^ In our study, the results indicated that the DFS and OS time in patients with low SII would have survival longer than those patients with high SII by lymph vessel invasion.

This study has several limitations. Firstly, this study is a retrospective single‐centre study and with the relatively low number of cases. More patients with breast cancer received NACT should be enrolled. Secondly, records on some parameters are not complete and lost to follow‐up. Thirdly, with the subgroup analysis, the numbers of patients are less and may influence the outcomes. Fourthly, according to use different cut‐off points and end‐points, and it is difficult to compare our results of SII with those of other studies. Further prospective and well‐designed randomized controlled trials are needed to support our findings, and the SII may be used in combination with other biomarkers to help predict clinical outcome in patients with breast cancer and is adopted in routine practice.

## 5 CONCLUSIONS

SII is the significant prognostic factor for patients with breast cancer and can effectively predict the survival in patients with breast cancer receiving NACT. It is very important to consider the high incidence of breast cancer and the unbalanced distribution of medical condition in China and that reproducible, conveniently and non‐invasive biomarkers should be applied to the prevention and treatment of breast cancer. It is very critical and meaningful to understand of haematological parameters and look for new targets for subjective treatment.

## CONFLICT OF INTEREST

The authors declare no competing financial interests.

## AUTHOR CONTRIBUTIONS

Li Chen wrote the original draft of the manuscript and reviewed and edited the manuscript; Xiangyi Kong involved in formal analysis; Zhongzhao Wang curated the data; Xiangyu Wang investigated the study; Yi Fang contributed to methodology and supervised the study; Jing Wang provided resources, acquired funding and involved in project administration.

## Data Availability

The material supporting the conclusion of this article has been included within the article.
